# Food Allergens in Ultra-Processed Foods According to the NOVA Classification System: A Greek Branded Food Level Analysis

**DOI:** 10.3390/nu15122767

**Published:** 2023-06-16

**Authors:** Alexandra Katidi, Stefania Xanthopoulou, Antonis Vlassopoulos, Stamoulis Noutsos, Kostas Priftis, Maria Kapsokefalou

**Affiliations:** 1 Laboratory of Chemistry and Food Analysis, Department of Food Science and Human Nutrition, Agricultural University of Athens, 11855 Athens, Greece; 2 Allergology and Pulmonology Unit, 3rd Pediatric Department, National and Kapodistrian University of Athens, 12462 Athens, Greece

**Keywords:** ultra-processed foods, food allergens, NOVA, branded food composition database

## Abstract

Ultra-processed foods’ (UPFs’) consumption has been positively linked to the presence of allergic symptoms, but it is yet unknown whether this is linked to their nutritional composition or allergen load. This study used the ingredient lists available in the Greek Branded Food Composition Database, HelTH, to classify foods (*n =* 4587) into four grades of food processing (NOVA1–4) according to the NOVA System. Associations between NOVA grades and the presence of allergens (as an ingredient or trace) were studied. Overall, UPFs (NOVA4) were more likely to contain allergens than unprocessed foods, NOVA1 (76.1% vs. 58.0%). However, nested analyses among similar foods showed that in >90% of cases, processing degree was not linked to allergens’ presence. Recipe/matrix complexity was more strongly linked to allergens’ presence with NOVA4 foods declaring 1.3 allergenic ingredients vs. 0.4 allergenic ingredients in NOVA1 foods (*p* < 0.01). Exposure to trace allergens was more common for NOVA4 than NOVA1 foods (45.4% vs. 28.7%), but the extent of contamination was similar (2.3 vs. 2.8 trace allergens). Overall, UPFs are more complex mixtures with higher numbers of allergens per food and are more prone to cross-contamination. However, indicating a food’s degree of processing is not sufficient to help identify allergen-free choices within the same subcategory.

## 1. Introduction

Food allergies have become a growing public health concern in the last 2 to 3 decades, especially in westernized countries [1]. This rising trend has been referred to as the “second wave” of the allergy epidemic [2], affecting, according to estimations, up to 10% of the population, mainly children [3].

Most allergy incidents have been associated with a short list of foods [3]. Milk, eggs, fish, crustaceans, tree nuts, peanuts, wheat, and soybeans are thought to account for 90% of food allergy reactions [4]. In Europe, there are 14 allergens recognized as the most common and potent causes of food allergies and intolerances, namely, cereals containing gluten, milk, eggs, nuts, peanuts, soybeans, fish, crustaceans, mollusks, celery, lupin, sesame, mustard, and sulfites [5]. These substances must be indicated on food labels when used as ingredients, according to European legislation [6]. Moreover, precautionary labels warn of the adventitious presence of these allergens (unintentional contamination by contact with other products during processing, storage, or shipping) [7,8,9].

Allergic symptoms such as asthma, eczema, and wheezing have been correlated with a poor diet and the excessive consumption of ultra-processed foods (UPFs) [1,10,11,12,13]. It is hypothesized that UPFs contain more allergens than their less-processed counterparts [14]. Moreover, since UPFs are primarily produced in industrial facilities [15], cross-contamination with allergens appears to be more likely. Considering the numerous dietary restrictions linked to living with allergies, in terms of food choices [12], it is important to understand whether food processing is a facilitator or a barrier in their food choices.

UPFs’ consumption has been adversely linked not only with allergic symptoms but with human health overall [1,11,12,13,16,17,18,19,20,21,22,23,24]. The main mechanism linking UPFs’ consumption to allergic symptoms, to date, is linked to the nutritional composition of the UPF-rich diet [10,11]. However, a more obvious mechanism would be a higher risk of exposure to allergens either as a trace or an ingredient or exposure to secondary allergens (i.e., exposure to soy for individuals diagnosed with a peanut allergy).

Despite the interest in UPFs’ consumption and human health for such associations to be studied, systems need to be in place to categorize foods in terms of their extent, intensity, and methods of processing. The NOVA Classification System has been proposed and widely used [15,25,26,27] to categorize foods in NOVA1, NOVA2, NOVA3, and NOVA4 according to their level of processing, from “unprocessed or minimally processed foods” as NOVA1 to “UPFs” as NOVA4. According to NOVA (a name, not an acronym), UPFs consist of more complex mixes of ingredients [15]. The NOVA System tracks elements such as the presence of food extracts, additives, antioxidants, stabilizers, emulsifiers, and food ingredients that have been produced via industrial level processing (e.g., extruded ingredients, isolates, hydrolyzed ingredients, etc.) in order to assign foods to groups. It follows that, in order for foods to be properly assigned to NOVA Groups, researchers need to ensure access to a detailed product description, a complete ingredient list, and an indication of whether a food is industrially formulated or not [28]. Such information is not available in traditional food composition databases and access to branded food composition databases is needed to obtain such detailed information [7,14,29,30].

In this paper, we hypothesized that a higher allergen load could be a mechanism, beyond the nutritional composition, linking UPFs to allergic symptoms. To our best knowledge, no study to date has been conducted to assess whether the level of food processing, as estimated by the NOVA Classification System, is related to the presence and number of major food allergens. A potential explanation for this could be linked to the product information needed for both allergen identification and the NOVA Classification to be performed appropriately. New advances linked to the development of branded food composition databases allow for such detailed investigations to take place as they provide granular data of food composition at the ingredient level.

Previous studies have examined the prevalence of allergens in branded foods without considering the degree of processing for each food [7,14]. In contrast, the current analysis takes into consideration that not all packaged foods undergo the same degree of processing, hypothesizing that this may have an impact on their allergen load. In order to correctly classify foods based on their degree of processing using the NOVA System, one needs to have access to each food’s ingredient list. Similarly, the ingredient list is the sole reliable source for allergen identification. Although this question has already been posed, the lack of access to the ingredient lists in the previous investigations may alter the associations between processing and allergen presence. The current study aims to revisit this question utilizing a robust methodology and a suitable dataset that provides all the data needed.

Therefore, the current study uses such a branded food composition database to identify allergens and perform NOVA Classifications. This study aims to (i) estimate the prevalence of allergens among different NOVA Groups per food subcategory, (ii) investigate the variability of allergens in each NOVA Group per food subcategory, and (iii) test whether processing, as assessed by the NOVA Classification System, is associated with the introduction of allergens as an ingredient or as a contaminant per food subcategory.

## 2. Materials and Methods

### 2.1. Data Source

For the purpose of this study, the Hellenic Food Thesaurus, the Greek Branded Food Composition Database (BFCD) [31], was used. HelTH dynamically collects and curates food data as shown on-pack through online sampling of foods available in the main online supermarkets in Greece. From 2019 until now, HelTH has been expanded twice to include plant-based meat and dairy imitations, as well as pulses [32,33]. The last version of HelTH, used in the current analysis, included data for 4851 branded food products sold in Greece from November 2019 to October 2022.

HelTH contains comprehensive information on the ingredient lists, which allowed the researchers to classify foods based on the NOVA System and examine the presence of allergens [32,33]. From the 4851 products available in HelTH, products that lacked an image with a readable ingredient list were removed from the analysis (*n =* 27). An additional *n =* 237 products were excluded because the researchers did not have access to images from all sides of the product’s package. This step is especially relevant for products with precautionary statements for allergens as there is no regulated position on the packaging for such statements and they can be included on any side of the packaging [7]. Thus, *n =* 4587 products with complete allergens data were included in the study herein.

### 2.2. Food Allergens in the HelTH BFCD

As previously described [7], allergen prevalence was studied using branded data from HelTH for the 14 allergens required by European regulation [8] and arranged based on potential exposure risk [7,8]:

(A) “Allergen declared as an ingredient”; the allergen is explicitly included in the ingredient list or a statement such as “contains” follows the ingredient list [9].

(B) “Allergen declared in a precautionary statement”; the label mentions the allergen with a statement such as “may contain traces of”, “manufactured in a facility that also processed”, “may be present”. Adventitious presence and presence of traces were grouped together.

For the purpose of the current study, an additional variable was created to calculate the percentage of allergen-free products. For that, any food that contained an allergen either as an ingredient or as a precautionary statement was considered as a food containing allergen(s).

### 2.3. The NOVA Classification System

NOVA classifies foods, according to the level of processing, in the following four distinct groups [15]:

NOVA1, unprocessed or minimally processed foods: all edible plant or animal parts that have undergone no or minimal processing/preservation.

NOVA2, culinary ingredients: salt, sugar, oils, and starch that have been produced by traditional techniques from NOVA1 foods and are not added to another food recipe/matrix.

NOVA3, processed foods: foods that are processed with traditional techniques and usually combine multiple ingredients, at least one of which is a NOVA2 food.

NOVA4, UPFs: ready to eat or to heat foods, industrially formulated typically with five or more and usually many ingredients They also commonly include additives and extracts from other foods and contain small amounts of intact NOVA1 foods [15,30].

Based on the above definitions, the full product description, the ingredient list to identify additives, and the indicator of whether the food was industrially formulated were studied to classify foods into NOVA Groups. HelTH provides access to the aforementioned information and ingredient lists. Ingredients such as caloric and/or non-caloric sweeteners, added sodium, added oils, protein isolates or concentrates, added natural flavors, flavor enhancers, emulsifiers, bulking agents, thickeners, antioxidants, and added vitamins/minerals were considered NOVA4 ingredients and the foods were classified accordingly [15,28,30]. For the NOVA Classification, foods without a readable ingredient list (as mentioned for the allergens) were excluded (*n =* 27) [15,28,30].

### 2.4. Statistical Analysis

Statistical analysis was carried out using IBM SPSS Statistics^®^ (version 23, Northridge, CA, USA). Descriptive statistics were used to estimate the prevalence of allergens, allergens used as ingredients, and allergens used in a precautionary statement per NOVA Group of each food subcategory. Differences in the prevalence of allergens between different NOVA Groups of a food subcategory were tested using the chi-square test. Statistical significance was set at 0.01%.

## 3. Results

### 3.1. Prevalence of Allergens in Branded Foods per NOVA Groups

In total, 72.0% (*n =* 3301) of the branded food products declared at least one allergen either in the ingredient list or as a precautionary statement. Moreover, 71.4% (*n =* 3275) were classified as UPFs (NOVA4) by the NOVA System. Overall, 58.0% (*n =* 523) of NOVA1 foods, 47.8% (*n =* 33) of NOVA2, 73.8% (*n =* 253) of NOVA3, and 76.1% (*n =* 2492) of NOVA4 foods contained at least one allergen in their ingredient list or as a precautionary statement (*p* < 0.01). Table 1 presents the prevalence of allergens per NOVA group within different food subcategories. Differences in the prevalence of allergens among different NOVA Groups of the same food subcategory were detected in 4 out of the 41 subcategories included in the current study (*p* < 0.01). In particular, UPFs (NOVA4) presented a higher prevalence of allergens than their less-processed counterparts (NOVA1–3) in the “Rice or similar products”, “Non-chocolate confectionery or other sugar product”, “Juice or nectar”, and “Spice, Condiment or other Ingredient” food subcategories.

### 3.2. Number of Different Allergens in NOVA Groups

**Figure 1:**
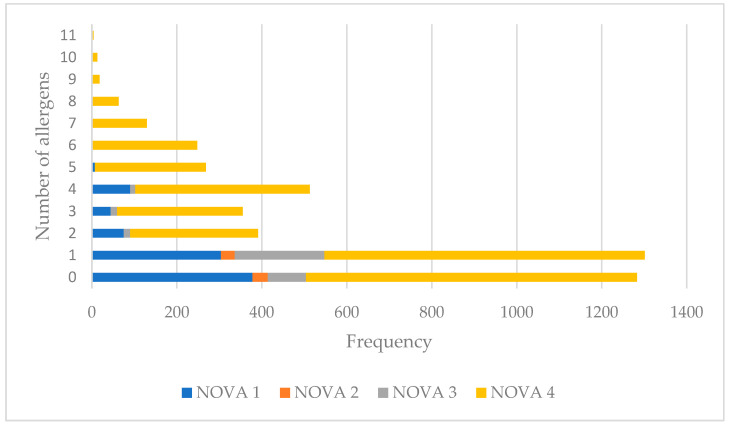
Number of allergens per NOVA Group.

Figure 1 presents the number of allergens included either as an ingredient or as a precautionary statement, per product in the four distinct NOVA Groups. On average, NOVA1 food products contained 1.1 allergens, NOVA2 0.5, NOVA3 1.0, while NOVA4 foods contained 2.6 allergens (*p* < 0.01). NOVA1 foods contained from 1 (34%, *n =* 304) to 9 different food allergens (0.1%, *n =* 1), NOVA2 contained 1 (46%, *n =* 32) or 3 allergens (1%, *n =* 1), NOVA3 products contained from 1 (61%, *n =* 211) to 4 allergens (3.5%, *n =* 12) in descending frequencies, and NOVA4 foods contained from 1 (23%, *n =* 754) to 11 allergens (0.1%, *n =* 4), either in the ingredient list or as a precautionary statement.

Supplementary Table S1 presents a nested analysis of the prevalence of the 14 allergens per NOVA Group and per food subcategory. Overall, among all UPFs (NOVA4), a larger number of different allergens was declared compared to their less-processed counterparts (NOVA1–3) in 17 out of the 22 food subcategories (Table S1).

Particularly for yogurts and cheese, among all non-ultra-processed foods (NOVA1 and NOVA3), only one allergen (milk) was identified, while among UPFs, eight different potential allergens were identified among yogurts (milk, gluten, soy, eggs, nuts, peanuts, and sesame) and the same was true among cheeses (milk, gluten, soy, eggs, mustard, sulphites, fish, and celery) (Table S1). For “milk imitation products”, three potential allergens were identified among NOVA1–3 foods (gluten, soy, nuts), but among NOVA4 foods, the potential allergens were nine (milk, gluten, soy, eggs, nuts, peanuts, sesame, mustard, and celery).

Along the same lines, in the “Nut or seed product” subcategory, NOVA1 foods could carry declarations for only one allergen (sesame), while among NOVA4 foods, the potential allergens were seven (milk, gluten, soy, nuts, peanuts, sesame, and sulphites). In the “Nuts” food subcategory, five potential allergens (gluten, soya, nuts, peanuts, and sesame) were found in the NOVA1 foods, while eight potential allergens (milk, gluten, soy, nuts, peanuts, sesame, mustard, and sulphites) were found in the NOVA4 foods. In the NOVA4 “Spices, Condiment or other ingredient” foods, all the 14 different allergens could be found, whereas in their less-processed counterparts (NOVA1–3), there were three potential allergens (Table S1).

In NOVA3 “Non-chocolate confectionery or other sugar product”, four different potential allergens (nuts, peanuts, sesame, sulphites) were found, while in the NOVA4 group, this number rose to eight (milk, gluten, soy, nuts, peanuts, sesame, eggs, sulphites). Among ultra-processed “Vegetables” (NOVA4), four potential allergens were found (milk, gluten, sulphites, celery), while among the NOVA1 foods, the only potential allergen was celery, and that was found in one product only.

This trend was reversed in the “Pasta and similar products” subcategory. For this subcategory, among NOVA4 foods, seven potential allergens were found (milk, gluten, soy, eggs, mustard, celery, lupin), while among NOVA1 foods, 10 potential allergens (milk, gluten, soy, eggs, nuts, peanuts, sesame, mustard, celery, lupin) could be found (Table S1).

### 3.3. Presence of Allergens as Ingredient per NOVA Group

A separate analysis was conducted to further specify whether this trend of higher allergen presence among NOVA4 foods described above was due to UPFs being more complex food recipes/matrixes with a higher number of ingredients and hence more allergens in their ingredient list.

**Figure 2:**
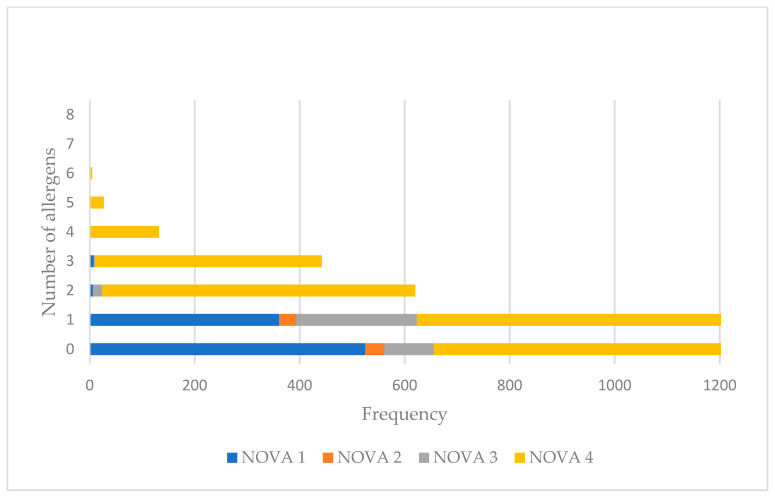
Number of allergens used as an ingredient per NOVA Group.

Figure 2 presents the number of allergens used as ingredients per product in the four distinct NOVA Groups. On average, NOVA1 foods carried 0.4 allergens as an ingredient, NOVA2 contained 0.5 allergens, while NOVA 3 and NOVA4 foods contained 0.8 and 1.3 allergens, respectively. Specifically, NOVA1 foods contained from one (40%, *n =* 361) to five food allergens (0.1%, *n =* 1) in their ingredient list, NOVA2 products contained one (48%, *n =* 33), NOVA3 from one (67%, *n =* 229) to three allergens (0,6%, *n =* 2) in descending frequencies, and NOVA4 foods contained from one (34%, *n =* 1125) to eight allergens (0.03%, *n =* 1) in descending frequencies.

Supplementary Table S2 presents a nested analysis of the prevalence of the 14 allergens used as ingredients per NOVA Group and per food subcategory. Overall, among all UPFs (NOVA4), a larger number of different allergens was declared compared to their less-processed counterparts (NOVA1–3) in 16 out of the 22 food subcategories (Table S2).

Particularly for yogurts and cheese, among all non-ultra-processed foods (NOVA1 and NOVA3), only one allergen (milk) was identified in their recipe, while among UPFs, five and six different potential allergens were identified in the recipes among yogurts and cheeses, respectively (Table S2). For “milk imitation products”, three potential allergens were identified in the ingredient list among NOVA1–3 foods, but among NOVA4 foods, the potential allergens in the ingredient list were seven.

NOVA1 “Rice or other grain” and NOVA1 and NOVA3 “Breakfast cereals” contained one allergen in their ingredient list (gluten), while their NOVA4 counterparts contained seven different allergens as part of their recipe. In NOVA4 “Nuts”, seven different allergens could be identified among the ingredients (milk, gluten, soy, nuts, peanuts, sesame, and sulphites), whereas among the NOVA1 “Nuts”, the potential allergens in the ingredients were only gluten and nuts. Similarly, among NOVA1 “Nut or Seed Products”, only sesame could be identified in the ingredient list, while among NOVA4 foods, a variety of five allergens could be identified in the ingredient list. Furthermore, the NOVA4 “Spices and Condiments” had the highest variability in potential allergens in the ingredient lists, as 12 allergens could be identified (Table S2).

### 3.4. Presence of Allergens as a Precautionary Statement per NOVA Group

A further analysis was conducted focusing only on the allergens declared as a precautionary statement. Regarding the prevalence of the 14 allergens used as a precautionary statement, overall, 38.8% of foods (*n =* 1781) declared at least one allergen. Twenty-nine percent of NOVA1 foods (*n =* 259), and 1.4% (*n =* 1) and 10.2% (*n =* 35) of NOVA2 and NOVA3 foods, respectively, contained allergen(s) as a precautionary statement, while in NOVA4 foods, traces of allergens were present in 45.4% (*n =* 1486).

**Figure 3:**
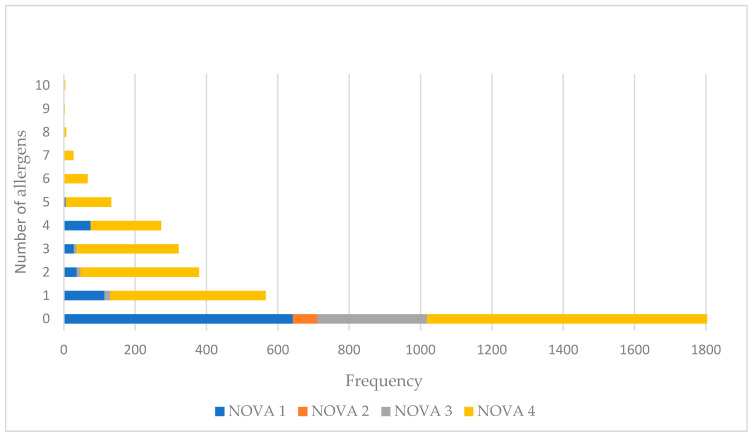
Number of allergens used as precautionary statement per NOVA Group.

Figure 3 presents the number of food allergens declared as a precautionary statement per product in the four distinct NOVA Groups. On average, NOVA1 foods contained 2.3 allergens as a precautionary statement, NOVA2 contained 2 allergens, and NOVA3 1.8, while NOVA4 contained 2.8 allergens. NOVA1 foods contained from 1 (44%, *n =* 114) to 6 (0.6%, *n =* 1) food allergens as a precautionary statement, in descending frequencies. NOVA4 foods contained from 1 (29.3%, *n =* 436) to 10 allergens as a precautionary statement (0.3, *n =* 4) in descending frequencies.

Supplementary Table S3 presents a nested analysis of the prevalence of the 14 allergens used as a precautionary statement per NOVA Group and per food subcategory. Overall, UPFs (NOVA4) carried a greater number of food allergens than their less-processed counterparts (NOVA1–3) in 16 out of the 22 food subcategories that included at least 2 different NOVA Groups (Table S3).

Among yogurts in particular, NOVA1 foods did not contain any allergens as a precautionary statement (Table S3). In contrast, in NOVA4 yogurts, precautionary statements for 7 different potential allergens were identified (gluten, soy, eggs, nuts, peanuts, sesame, and lupin). Among cheeses, NOVA3 cheese products did not carry allergen traces, while NOVA4 cheeses carried precautionary statements for 6 different potential allergens (gluten, soy, eggs, mustard, sulphites, and celery) (Table S3). Among “Milk imitation products”, NOVA1 and NOVA3 foods declared 1 (nuts) and 2 (soy and nuts) different allergens as a precautionary statement, respectively, while among NOVA4 foods, 9 different allergens were declared as a precautionary statement (milk, gluten, soy, eggs, nuts, peanuts, sesame, mustard, and celery). In the “Rice and similar product” subcategory, NOVA1 products declared 6 allergens as a precautionary statement, while NOVA4 products declared 11 different allergens as a precautionary statement. In the “Breakfast cereals” subcategory, 5 different allergens were identified as a precautionary statement among the NOVA1 products, while among the NOVA4 breakfast cereals, 9 different allergens were found declared as a precautionary statement (Table S3).

## 4. Discussion

This study uses one of the few available branded food composition databases to assess the association between the degree of processing and allergen presence in packaged foods sold in Greece. With access to granular food composition data on ingredients, BFCDs are the ideal tools to ensure high precision in NOVA food classification in terms of the degree of processing and allergen presence.

Based on the information provided in the ingredient list and the manufacturer declarations, 72% of all foods contained at least one allergen and 71.4% of all foods were classified as UPFs under the NOVA System. Overall, NOVA4 foods were 50% more likely to carry an allergen declaration than NOVA1 (minimally processed) foods, but when the analysis was focused among similar foods with different degrees of processing, the association between NOVA and allergens’ presence could no longer be observed.

Namely, differences in the prevalence of allergens according to the degree of processing was seen for “Spice, Condiment or other Ingredient”, “Non-chocolate confectionery or other sugar product”, “Juice or Nectar”, and “Rice or Rice products”. However, these categories are heterogeneous in terms of the foods assigned to them. This heterogeneity is linked to the fact that although foods assigned to these categories have the same role in the consumers’ diet, their nature is different. Thus, NOVA4 foods in these categories are usually more complex food products both for their recipe development and for their matrixes (bouillons, risotto, Turkish delight, nougat, multi-fruit juices) compared to NOVA1 foods (desiccated coconut, plain rice, single fruit juices). Hence, the likelihood of allergens’ presence seems more probably linked to the product complexity rather than the degree of processing. Therefore, it is safe to stipulate that the presence of food allergens was primarily associated with the nature (e.g., milk, eggs) and complexity of the foods, and to a lower degree with the level of processing.

Although, the degree of processing may not be strongly linked with the likelihood of allergen presence, i.e., it is equally difficult to find allergen-free foods in all NOVA groups, the number of unique allergens present per food seems to be positively associated with processing. NOVA1 foods declared on average ~1 allergen per food and that was often the base ingredient for the food category (milk for dairy, gluten for cereal-based foods), while NOVA4 foods declared on average 2.6 allergens per food. Usually, those additional allergens were again linked to the complexity of the food recipe/matrix in NOVA4 foods. For instance, in NOVA4 yogurts, except for the expected presence of milk allergens, gluten, soy, eggs, nuts, peanuts, sesame, and lupin could also be found either as an ingredient or as a precautionary statement. Furthermore, fish was found as an ingredient in a NOVA4 cheese product, mollusks and crustaceans were mentioned in a precautionary statement of a cereal product, and lupin traces were declared on a yogurt’s label.

In fact, when allergens were studied separately based on whether they were an ingredient or a trace caused by cross-contamination, the association between allergens’ presence and processing differed substantially. Among NOVA1 foods, 41.7% contained at least one allergen in the ingredient list with an average content of 0.4 allergens per food. On the other hand, 70.8% of NOVA4 foods contained allergens in their ingredients with an average content of 1.3 allergens per food. This is indicative of allergens’ presence primarily linked to the nature of the food for NOVA1 foods and food complexity for NOVA4 foods.

The same conclusion could not easily be reached for the exposure of foods in allergen traces through cross-contamination, as 28.7% of NOVA1 foods declared trace elements of allergens with an average content of 2.3 potential allergens per food, and 45.4% of NOVA4 foods declared trace elements of allergens with an average content of 2.8 potential allergens per food. A potential explanation for this could be that it is the packaging and manufacturing process that is linked to cross-contamination and not the degree of processing per se. These hypotheses are also supported by previous research showing that indeed NOVA4 foods tend to be composite formulations [24] indicative of the wider variety of food allergens in their ingredient lists. In addition, accidental cross-contamination in industrial facilities with multiple products is a known source of the declaration of multiple allergens in the precautionary statements [34]. At this point, it is important to highlight that processing could also be linked to the elimination of specific allergens in food products that address the specific needs of allergic persons. For example, milk was present in all (100%) dairy foods irrespective of the degree of processing, but only 1.7% of NOVA4 dairy imitations included milk. Similarly, 98.2% of NOVA1 pasta foods and 100% of NOVA1 breakfast cereals included gluten. At the same time, 84.6% of NOVA4 pasta foods and 91.7% of NOVA4 breakfast cereals included gluten, highlighting the capacity for targeted allergen removal through food processing.

To date, the literature has focused on the association of UPFs’ consumption (usually measured through the NOVA System) with food allergies and their symptoms. Although research suggests a positive association between the two [10,11,12,13,35], it is unclear whether the mechanism of action is that of higher exposure to allergens or linked to the nutritional quality of UPFs and the nutritional adequacy of UPF-rich diets. Previous studies have suggested a positive association of UPFs’ consumption with asthma and wheezing in children and adolescents; however, both the presence of additives and the positive energy balance linked to UPFs’ consumption have been proposed as mediators [10,11,35,36]. In fact, an analysis of 2190 cases of childhood asthma and wheezing showed that energy intake adjustments explained the association between UPFs’ consumption and symptoms [36]. Similarly, the consumption of UPFs has been associated with a lower intake of proteins and a higher intake of total fat and sugar [37] with an unknown impact on food allergy symptoms [11].

In the current analysis, we aimed to highlight a new potential element of processing and the severity of allergic symptoms. According to the literature, it is possible that people living with food allergies develop sensitivity to other foods with homologous proteins [38]. For instance, mammalian milks, eggs, fish, and shellfish present high cross-reactivity [34]. Approximately 5% of children with peanut allergies may also react to soy and other legumes [38], as their allergenic ingredients present similarities [34]. Usually, people sensitized to one tree nut show cross-reactivity to other tree nuts too. In this light, exposure to UPFs, even if free of a specific allergen, could be linked with exposure to secondary allergens, even in minuscule amounts, which in turn could hinder the allergy management process. Based on the current findings, one might be prompted to assume that individuals with multiple food allergies should be extra cautious while consuming UPFs. This conclusion, although partially justified, is not entirely useful as guidance.

The current study is focused on understanding whether processing is a barrier (e.g., presence of unexpected allergens, cross-contamination) or a facilitator (e.g., gluten-free products, lactose-free products, etc.) for people living with allergies. It is important to note that while previous studies have suggested that food processing may reduce the allergenicity of foods [39,40], the scope of this cross-sectional study is to investigate the differences in the presence of allergens in processed and non-processed products. Taken altogether, the findings indicate that the degree of processing is likely to be associated with a higher ingredient complexity and hence a larger number of potential allergens present per food. These allergens could be linked to the base ingredient, but they could also be introduced as additional ingredients. Prompting individuals to avoid UPFs as declared under the NOVA System may not be a straightforward and easily executable guidance. People living with allergies should be trained to read ingredient lists and be cautious with their food choices irrespective of the degree of processing, as allergen-free foods are rare in all NOVA groups. At the same time, individuals should not assume that guessing allergen presence from the product name is a sufficient measure, and they should pay attention to ingredient declarations and precautionary statements, especially when they purchase complex foods. A blanket statement against UPFs for those living with allergies could actually create a further burden in their available food choices as they would avoid foods specifically processed to exclude a given allergen.

In particular, this study shows that the degree of food processing, as estimated by the NOVA Classification System, is not sufficient to help consumers identify allergen-free choices within the same subcategory. In addition, it should be considered that food processing is a necessary means for the development of new products specifically designed for people living with allergies. For instance, more than 90% of allergen-free claimed foods (gluten-free, dairy-free, etc.) are characterized as UPFs by the NOVA Classification (data not shown). Therefore, in some cases, it may be more functional to focus on the formulation and nutritional quality of a food product, rather than its degree of processing.

At this point, it is important to mention that this study also presents some limitations. Firstly, HelTH as a BFCD includes information from the labels of prepackaged products. Thus, foods mainly sold in a fresh form, without food labels, cannot be mapped by HelTH, resulting in an underrepresentation of the foods available in the market. Secondly, prepackaged foods typically undergo more processing than their fresh counterparts, which may lead to an overestimation of the NOVA4 Group for each food subcategory [41]. Furthermore, data were collected from food labels that pose the risk of undeclared allergens [7], linked to a potential allergen underestimation. It is worth mentioning that this cross-sectional study investigated the association of the degree of processing and the presence of the most common food allergens according to EU legislation, by mapping data from branded food products. Future studies should aim to illuminate the link between food processing and allergenic ingredients by measuring the quantity as well as the allergenicity of them and assess whether UPFs are positively linked to allergies.

Nonetheless, this work offers evidence for the presence of food allergens in UPFs, and more specifically, in similar branded foods with a different degree of processing. Past studies that assessed the degree of processing have not always used branded data for nutritional assessment, and thus, ingredient lists were not available to characterize UPFs [10,36,42,43,44]. When aiming to classify foods appropriately into NOVA Groups, access to a product’s ingredient list is necessary. According to the NOVA Classification System, the presence of additives and other ingredients that cannot be found in a kitchen can classify a food into the NOVA4 Group. Therefore, according to the NOVA Classification System, UPFs not only belong to specific food groups such as extruded savory snacks, sweet biscuits, preserved meat, etc., but can also be found among plain yogurt desserts, milk products, prepacked fruit, vegetable products, etc. Branded food datasets provide detailed product information, making them a particularly important tool for accurately categorizing foods according to the NOVA Classification and to further advance questions on public health concerns related to UPFs.

## 5. Conclusions

This study is the first to investigate the association between the level of processing and allergen presence in branded foods, using a database that provides detailed and granular information about each food’s ingredients. Using the appropriate data, foods were classified in NOVA categories and allergens were tracked separately as an ingredient and a trace. Overall, UPFs (NOVA4) are, according to the NOVA Classification System, more complex mixtures, and also present higher numbers of allergens per food and are more prone to cross-contamination. However, as indicated by the analysis of combined ingredient or trace allergen content, the degree of processing alone is not sufficient to guide consumer choices in order to avoid allergen exposure, and ingredient lists should be consulted in all cases.

## Figures and Tables

**Table 1:** Prevalence of allergens in branded foods, per NOVA Group and food subcategory.

Food Category	Food Subcategory	NOVA Group	Prevalence of Allergens *n* (%)	*p*-Value
Milk, milk product, or milk substitute (*n* = 965)	Milk (*n* = 171)	NOVA1 (*n* = 94)	94 (100)	n.a.
		NOVA3 (*n* = 6)	6 (100)	
		NOVA4 (*n* = 71)	71 (100)	
	Yogurt (*n* = 168)	NOVA1 (*n* = 46)	46 (100)	n.a.
		NOVA4 (*n* = 122)	122 (100)	
	Cheese (*n* = 209)	NOVA3 (*n* = 124)	124 (100)	n.a.
		NOVA4 (*n* = 85)	85 (100)	
	Milk substitute (*n* = 334)	NOVA1 (*n* = 6)	6 (100)	0.400
		NOVA3 (*n* = 37)	30 (81.1)	
		NOVA4 (*n* = 291)	227 (78.0)	
	Milk cream (*n* = 40)	NOVA4 (*n* = 40)	40 (100)	n.a.
	Dairy dessert (*n* = 43)	NOVA4 (*n* = 43)	43 (100)	n.a.
Fresh or processed eggs (*n* = 36)	Fresh or processed eggs (*n* = 35)	NOVA1 (*n* = 34)	34 (100)	n.a.
		NOVA4 (*n* = 1)	1 (100)	
	Egg imitation (*n* = 1)	NOVA4 (*n* = 1)	1 (100)	n.a.
Meat or related product (*n* = 248)	Poultry meat (*n* = 2)	NOVA4 (*n* = 2)	2 (100)	n.a.
	Preserved meat (*n* = 82)	NOVA1 (*n* = 1)	1 (100)	0.013
		NOVA3 (*n* = 2)	0 (0.0)	
		NOVA4 (*n* = 79)	65 (82.3)	
	Sausage or similar meat (*n* = 37)	NOVA4 (*n* = 37)	26 (70.3)	n.a.
	Meat dish (*n* = 17)	NOVA4 (*n* = 17)	16 (94.1)	n.a.
	Meat analogue (*n* = 110)	NOVA4 (*n* = 110)	89 (80.9)	n.a.
Seafood or related product (*n* = 78)	Seafood product (*n* = 78)	NOVA3 (*n* = 52)	52 (100)	n.a.
		NOVA4 (*n* = 26)	26 (100)	
Fat or oil (*n* = 84)	Vegetable fat or oil (*n* = 8)	NOVA2 (*n* = 1)	0 (0.0)	0.408
		NOVA4 (*n* = 7)	3 (42.9)	
	Margarine or lipid of mixed origins (*n* = 39)	NOVA4 (*n* = 39)	33 (84.6)	n.a.
	Butter or other animal fat (*n* = 37)	NOVA2 (*n* = 32)	32 (100)	n.a.
		NOVA4 (*n* = 2)	2 (100)	
Grain or grain product (*n* = 1104)	Cereal or cereal-like milling products (*n* = 51)	NOVA4 (*n* = 51)	51 (100)	n.a.
	Rice or similar products (*n* = 97)	NOVA1 (*n* = 64)	8 (12.5)	<0.01
		NOVA3 (*n* = 2)	0 (0.0)	
		NOVA4 (*n* = 31)	28 (90.3)	
	Pasta and similar products (*n* = 200)	NOVA1 (*n* = 165)	163 (98.8)	0.082
		NOVA3 (*n* = 9)	9 (100)	
		NOVA4 (*n* = 26)	24 (92.3)	
	Breakfast cereals (*n* = 149)	NOVA1 (*n* = 4)	4 (100)	0.983
		NOVA3 (*n* = 1)	1 (100)	
		NOVA4 (*n* = 144)	143 (99.3)	
	Bread and similar products (*n* = 242)	NOVA4 (*n* = 242)	241 (99.6)	n.a.
	Fine bakery ware (*n* = 279)	NOVA4 (*n* = 279)	279 (100)	n.a.
	Savory cereal dish (*n* = 86)	NOVA4 (*n* = 86)	86 (100)	n.a.
Nut, seed, or kernel (*n* = 127)	Nuts (*n* = 65)	NOVA1 (*n* = 20)	20 (100)	n.a.
		NOVA3 (*n* = 14)	14 (100)	
		NOVA4 (*n* = 31)	31 (100)	
	Seeds and kernels (*n* = 35)	NOVA3 (*n* = 18)	0 (0.0)	0.062
		NOVA4 (*n* = 17)	3 (17.6)	
	Nut or seed product (*n* = 27)	NOVA1 (*n* = 9)	9 (100)	n.a.
		NOVA4 (*n* = 18)	18 (100)	
Vegetable or vegetable product (*n* = 535)	Vegetable (excluding potato) (*n* = 170)	NOVA1 (*n* = 65)	1 (1.5)	0.043
		NOVA3 (*n* = 35)	4 (11.4)	
		NOVA4 (*n* = 70)	9 (12.9)	
	Starchy root or potato (*n* = 21)	NOVA1 (*n* = 1)	1 (100)	0.058
		NOVA3 (*n* = 2)	0 (0.0)	
		NOVA4 (*n* = 18)	13 (60.1)	
	Pulse or pulse product (*n* = 341)	NOVA1 (*n* = 341)	136 (39.9)	n.a.
Fruit or fruit product (*n* = 42)	Processed fruit product (*n* = 42)	NOVA1 (*n* = 1)	1 (100)	0.078
		NOVA3 (*n* = 5)	0 (0.0)	
		NOVA4 (*n* = 36)	16 (44.4)	
Sugar or sugar product (*n* = 404)	Sugar, honey, or syrup (*n* = 46)	NOVA1 (*n* = 1)	0 (0.0)	n.a.
		NOVA2 (*n* = 35)	0 (0.0)	
		NOVA3 (*n* = 6)	0 (0.0)	
		NOVA4 (*n* = 4)	0 (0.0)	
	Jam or marmalade (*n* = 83)	NOVA4 (*n* = 83)	17 (20.5)	n.a.
	Non-chocolate confectionery or other sugar product (*n* = 68)	NOVA3 (*n* = 22)	10 (45.5)	<0.01
		NOVA4 (*n* = 46)	45 (97.8)	
	Chocolate or chocolate product (*n* = 207)	NOVA4 (*n* = 207)	207 (100)	n.a.
Beverage (*n* = 446)	Juice or nectar (*n* = 163)	NOVA1 (*n* = 48)	0 (0.0)	<0.01
		NOVA3 (*n* = 1)	1 (100)	
		NOVA4 (*n* = 114)	2 (1.8)	
	Non-alcoholic beverage (*n* = 283)	NOVA4 (*n* = 283)	17 (6.0)	n.a.
Miscellaneous food product (*n* = 446)	Spice, condiment, or other ingredient (*n* = 282)	NOVA1 (*n* = 1)	0 (0.0)	0.009
		NOVA2 (*n* = 1)	1 (100)	
		NOVA3 (*n* = 7)	2 (28.6)	
		NOVA4 (*n* = 273)	208 (76.2)	
	Prepared food product (*n* = 164)	NOVA4 (*n* = 164)	146 (89.0)	n.a.
Ready meals (*n* = 78)	Ready to eat food (*n* = 38)	NOVA4 (*n* = 38)	36 (94.7)	n.a.
	Frozen, semi-ready meal (*n* = 40)	NOVA4 (*n* = 40)	20 (50.0)	n.a.

The confidence level was set at 99%. n.a.: comparison not applicable due to the presence of one NOVA Group in the food subcategory or due to the presence of allergens in all (100%) or none (0%) of the foods.

## Data Availability

HelTH is available at https://www.eurofir.org/our-tools/foodexplorer/ (accessed on 26 January 2023).

## Supplementary Materials

The following supporting information can be downloaded at: https://www.mdpi.com/article/10.3390/nu15122767/s1, Table S1: Prevalence of the 14 allergens in branded foods per NOVA Group of each food subcategory; Table S2: Prevalence of the 14 allergens in the products’ ingredient list per NOVA Group of each food subcategory; Table S3: Prevalence of the 14 allergens as traces in a precautionary statement per NOVA Group of each food subcategory.
