# Supplementary material for: Food Allergens in Ultra-Processed Foods According to the NOVA Classification System: A Greek Branded Food Level Analysis
Source: Nutrients. 2023 Jun 16;15(12):2767. doi: 10.3390/nu15122767 (PMC10302194; doi:10.3390/nu15122767)
Supplement: Supplementary file 1 [file nutrients-15-02767-s001.zip › nutrients-2392151-supplementary.pdf]

Table S1 Prevalence of the 14 allergens in branded foods per NOVA Group of each food subcategory.

Food Subcategory	NOVA Group	Milk	Gluten	Soy	Eggs	Nuts	Peanuts	Sesame	Mustard	Sulphites	Fish	Crustaceans	Mollusks	Celery	Lupin
Milk (n=171)	NOVA1 (n=94)	94 (100.0)	0 (0.0)	0 (0.0)	0 (0.0)	0 (0.0)	0 (0.0)	0 (0.0)	0 (0.0)	0 (0.0)	0 (0.0)	0 (0.0)	0 (0.0)	0 (0.0)	0 (0.0)
	NOVA3 (n=6)	6 (100.0)	0 (0.0)	0 (0.0)	0 (0.0)	0 (0.0)	0 (0.0)	0 (0.0)	0 (0.0)	0 (0.0)	0 (0.0)	0 (0.0)	0 (0.0)	0 (0.0)	0 (0.0)
	NOVA4 (n=71)	71 (100.0)	0 (0.0)	3 (4.2)	0 (0.0)	0 (0.0)	0 (0.0)	0 (0.0)	0 (0.0)	0 (0.0)	0 (0.0)	0 (0.0)	0 (0.0)	0 (0.0)	0 (0.0)
Yogurt (n=168)	NOVA1 (n=46)	46 (100.0)	0 (0.0)	0 (0.0)	0 (0.0)	0 (0.0)	0 (0.0)	0 (0.0)	0 (0.0)	0 (0.0)	0 (0.0)	0 (0.0)	0 (0.0)	0 (0.0)	0 (0.0)
	NOVA4 (n=122)	122 (100.0)	32 (26.2)	10 (8.2)	11 (9.0)	21 (17.2)	7 (5.7)	10 (8.2)	0 (0.0)	0 (0.0)	0 (0.0)	0 (0.0)	0 (0.0)	0 (0.0)	1 (0.8)
Cheese (n=209)	NOVA3 (n=124)	124 (100.0)	0 (0.0)	0 (0.0)	0 (0.0)	0 (0.0)	0 (0.0)	0 (0.0)	0 (0.0)	0 (0.0)	0 (0.0)	0 (0.0)	0 (0.0)	0 (0.0)	0 (0.0)
	NOVA4 (n=85)	85 (100.0)	4 (4.7)	3 (3.5)	7 (8.2)	0 (0.0)	0 (0.0)	0 (0.0)	4 (4.7)	5 (5.9)	1 (1.2)	0 (0.0)	0 (0.0)	4 (4.7)	0 (0.0)
Milk Substitute (n=334)	NOVA1 (n=6)	0 (0.0)	1 (16.7)	3 (50.0)	0 (0.0)	3 (50.0)	0 (0.0)	0 (0.0)	0 (0.0)	0 (0.0)	0 (0.0)	0 (0.0)	0 (0.0)	0 (0.0)	0 (0.0)
	NOVA3 (n=37)	0 (0.0)	13 (35.1)	7 (18.9)	0 (0.0)	15 (40.5)	0 (0.0)	0 (0.0)	0 (0.0)	0 (0.0)	0 (0.0)	0 (0.0)	0 (0.0)	0 (0.0)	0 (0.0)
	NOVA4 (n=291)	14 (4.8)	56 (19.2)	80 (27.5)	8 (2.7)	146 (50.2)	7 (2.4)	31 (10.7)	16 (5.5)	0 (0.0)	0 (0.0)	0 (0.0)	0 (0.0)	16 (5.5)	0 (0.0)
Milk Cream (n=40)	NOVA4 (n=40)	40 (100.0)	0 (0.0)	2 (5.0)	0 (0.0)	0 (0.0)	0 (0.0)	0 (0.0)	0 (0.0)	0 (0.0)	0 (0.0)	0 (0.0)	0 (0.0)	0 (0.0)	0 (0.0)
Dairy Dessert (n=43)	NOVA4 (n=43)	41 (95.3)	22 (51.2)	33 (76.7)	24 (55.8)	35 (81.4)	0 (0.0)	4 (9.3)	0 (0.0)	0 (0.0)	0 (0.0)	0 (0.0)	0 (0.0)	0 (0.0)	0 (0.0)
Fresh or processed eggs (n=35)	NOVA1 (n=34)	3 (8.8)	3 (8.8)	3 (8.8)	34 (100.0)	3 (8.8)	0 (0.0)	0 (0.0)	0 (0.0)	0 (0.0)	0 (0.0)	0 (0.0)	0 (0.0)	0 (0.0)	0 (0.0)
	NOVA4 (n=1)	0 (0.0)	0 (0.0)	0 (0.0)	1 (100.0)	0 (0.0)	0 (0.0)	0 (0.0)	0 (0.0)	0 (0.0)	0 (0.0)	0 (0.0)	0 (0.0)	0 (0.0)	0 (0.0)
Egg imitation (n=1)	NOVA4 (n=1)	0 (0.0)	0 (0.0)	1 (100.0)	0 (0.0)	0 (0.0)	0 (0.0)	1 (100.0)	1 (100)	0 (0.0)	0 (0.0)	0 (0.0)	0 (0.0)	1 (100)	0 (0.0)
Poultry meat (n=2)	NOVA4 (n=2)	2 (100.0)	0 (0.0)	0 (0.0)	0 (0.0)	0 (0.0)	0 (0.0)	0 (0.0)	0 (0.0)	0 (0.0)	0 (0.0)	0 (0.0)	0 (0.0)	0 (0.0)	0 (0.0)

Meat analogue (n=110)	NOVA4 (n=110)	0 (0.0)	70 (63.6)	60 (54.5)	3 (2.7)	10 (9.1)	0 (0.0)	18 (16.4)	43 (39.1)	3 (2.7)	0 (0.0)	0 (0.0)	0 (0.0)	39 (35.5)	4 (3.6)
Preserved meat (n=82)	NOVA1 (n=1)	1 (100.0)	0 (0.0)	1 (100.0)	1 (100).0	1 (100.0)	1 (100)	0 (0.0)	1 (100)	1 (100)	0 (0.0)	0 (0.0)	0 (0.0)	1 (100)	0 (0.0)
	NOVA3 (n=2)	0 (0.0)	0 (0.0)	0 (0.0)	0 (0.0)	0 (0.0)	0 (0.0)	0 (0.0)	0 (0.0)	0 (0.0)	0 (0.0)	0 (0.0)	0 (0.0)	0 (0.0)	0 (0.0)
	NOVA4 (n=79)	54 (68.4)	17 (21.5)	56 (70.9)	29 (36.7)	41 (51.9)	10 (12.7)	1 (1.3)	57 (72.2)	28 (35.4)	0 (0.0)	0 (0.0)	0 (0.0)	52 (65.8)	0 (0.0)
Sausage or similar meat (n=37)	NOVA4 (n=37)	21 (56.8)	6 (16.2)	20 (54.1)	13 (35.1)	11 (29.7)	5 (13.5)	0 (0.0)	20 (54.1)	12 (32.4)	0 (0.0)	0 (0.0)	0 (0.0)	19 (51.4)	0 (0.0)
Meat dish (n=17)	NOVA4 (n=17)	15 (88.2)	15 (88.2)	5 (29.4)	5 (29.4)	1 (5.9)	1 (5.9)	0 (0.0)	8 (47.1)	0 (0.0)	0 (0.0)	0 (0.0)	0 (0.0)	2 (11.8)	0 (0.0)
Seafood product (n=78)	NOVA3 (n=52)	0 (0.0)	1 (1.9)	2 (3.8)	0 (0.0)	0 (0.0)	0 (0.0)	0 (0.0)	0 (0.0)	0 (0.0)	50 (96.2)	1 (1.9)	2 (3.8)	3 (5.8)	0 (0.0)
	NOVA4 (n=26)	2 (7.7)	9 (34.6)	2 (7.7)	1 (3.8)	0 (0.0)	0 (0.0)	0 (0.0)	2 (7.7)	2 (7.7)	19 (73.1)	1 (3.8)	9 (34.6)	1 (3.8)	0 (0.0)
Vegetable fat or oil (n=8)	NOVA2 (n=1)	0 (0.0)	0 (0.0)	0 (0.0)	0 (0.0)	0 (0.0)	0 (0.0)	0 (0.0)	0 (0.0)	0 (0.0)	0 (0.0)	0 (0.0)	0 (0.0)	0 (0.0)	0 (0.0)
	NOVA4 (n=7)	3 (42.9)	0 (0.0)	0 (0.0)	0 (0.0)	0 (0.0)	0 (0.0)	0 (0.0)	0 (0.0)	0 (0.0)	0 (0.0)	0 (0.0)	0 (0.0)	0 (0.0)	0 (0.0)
Margarine or lipid of mixed origins (n=39)	NOVA4 (n=39)	33 (84.6)	0 (0.0)	1 (2.6)	0 (0.0)	0 (0.0)	0 (0.0)	0 (0.0)	0 (0.0)	0 (0.0)	0 (0.0)	0 (0.0)	0 (0.0)	0 (0.0)	0 (0.0)
Butter or other animal fat (n=34)	NOVA2 (n=32)	32 (100.0)	0 (0.0)	0 (0.0)	0 (0.0)	0 (0.0)	0 (0.0)	0 (0.0)	0 (0.0)	0 (0.0)	0 (0.0)	0 (0.0)	0 (0.0)	0 (0.0)	0 (0.0)
	NOVA4 (n=2)	2 (100.0)	0 (0.0)	0 (0.0)	0 (0.0)	0 (0.0)	0 (0.0)	0 (0.0)	0 (0.0)	0 (0.0)	0 (0.0)	0 (0.0)	0 (0.0)	0 (0.0)	0 (0.0)
Cereal or cereal-like milling products and derivatives (n=51)	NOVA4 (n=51)	36 (70.6)	51 (100.0)	27 (52.9)	35 (68.6)	15 (29.4)	4 (7.8)	21 (41.2)	16 (31.4)	1 (2.0)	1 (2.0)	1 (2.0)	1 (2.0)	5 (9.8)	0 (0.0)
Rice or other grain (n=97)	NOVA1 (n=64)	0 (0.0)	3 (4.7)	1 (1.6)	1 (1.6)	5 (7.8)	5 (7.8)	5 (7.8)	0 (0.0)	0 (0.0)	0 (0.0)	0 (0.0)	0 (0.0)	0 (0.0)	0 (0.0)

	NOVA3 (n=2)	0 (0.0)	0	0 (0.0)	0 (0.0)	0 (0.0)	0 (0.0)	0 (0.0)	0 (0.0)	0 (0.0)	0 (0.0)	0 (0.0)	0 (0.0)	0 (0.0)	0 (0.0)
	NOVA4 (n=31)	19 (61.3)	28 (90.3)	22 (71.0)	21 (67.7)	0 (0.0)	5 (16.1)	13 (41.9)	19 (61.3)	0 (0.0)	7 (22.6)	3 (9.7)	1 (3.2)	19 (61.3)	7 (22.6)
Pasta and similar products (n=200)	NOVA1 (n=165)	14 (8.5)	262 (98.2)	64 (38.8)	52 (31.5)	1 (0.6)	1 (0.6)	1 (0.6)	1 (0.6)	0 (0.0)	0 (0.0)	0 (0.0)	0 (0.0)	1 (0.6)	1 (0.6)
	NOVA3 (n=9)	8 (88.9)	9 (100.0)	0 (0.0)	6 (66.7)	0 (0.0)	0 (0.0)	3 (33.3)	0 (0.0)	0 (0.0)	1 (11.1)	1 (11.1)	0 (0.0)	0 (0.0)	0 (0.0)
	NOVA4 (n=26)	20 (76.9)	22 (84.6)	11 (42.3)	19 (73.1)	0 (0.0)	0 (0.0)	0 (0.0)	1 (3.8)	0 (0.0)	0 (0.0)	0 (0.0)	0 (0.0)	1 (3.8)	3 (11.5)
Breakfast cereals (n=149)	NOVA1 (n=4)	1 (25.0)	4 (100.0)	4 (100.0)	0 (0.0)	1 (25.0)	0 (0.0)	1 (25.0)	0 (0.0)	1 (25.0)	0 (0.0)	0 (0.0)	0 (0.0)	0 (0.0)	0 (0.0)
	NOVA3 (n=1)	0 (0.0)	1 (100.0)	1 (100.0)	0 (0.0)	1 (100.0)	1 (100)	0 (0.0)	0 (0.0)	0 (0.0)	0 (0.0)	0 (0.0)	0 (0.0)	0 (0.0)	0 (0.0)
	NOVA4 (n=144)	117 (81.3)	135 (93.8)	94 (65.3)	3 (2.1)	112 (77.8)	56 (38.9)	26 (18.1)	2 (1.4)	12 (8.3)	0 (0.0)	0 (0.0)	0 (0.0)	0 (0.0)	2 (1.4)
Bread and similar products (n=242)	NOVA4 (n=242)	120 (49.6)	238 (98.3)	73 (30.2)	88 (36.4)	47 (19.4)	4 (1.7)	149 (61.6)	4 (1.7)	4 (1.7)	0 (0.0)	0 (0.0)	0 (0.0)	2 (0.8)	3 (1.2)
Fine bakery ware (n=279)	NOVA4 (n=279)	240 (86.0)	277 (99.3)	215 (77.1)	197 (70.6)	217 (77.8)	68 (24.4)	146 (52.3)	5 (1.8)	20 (7.2)	0 (0.0)	0 (0.0)	0 (0.0)	5 (1.8)	20 (7.2)
Savory cereal dish (n=86)	NOVA4 (n=86)	85 (98.8)	86 (100.0)	45 (52.3)	66 (76.7)	37 (43.0)	2 (2.3)	65 (75.6)	40 (46.5)	2 (2.3)	2 (2.3)	0 (0.0)	0 (0.0)	24 (27.9)	1 (1.2)
Nuts (n=65)	NOVA1 (n=20)	0 (0.0)	19 (95.0)	1 (5)	0 (0.0)	20 (100.0)	18 (90.0)	13 (65.0)	0 (0.0)	0 (0.0)	0 (0.0)	0 (0.0)	0 (0.0)	0 (0.0)	0 (0.0)
	NOVA3 (n=14)	0 (0.0)	10 (71.4)	0 (0.0)	0 (0.0)	13 (92.9)	14 (100)	10 (71.4)	0 (0.0)	0 (0.0)	0 (0.0)	0 (0.0)	0 (0.0)	0 (0.0)	0 (0.0)
	NOVA4 (n=31)	4 (12.9)	28 (90.3)	5 (16.1)	0 (0.0)	31 (100.0)	30 (96.8)	21 (67.7)	1 (3.2)	4 (12.9)	0 (0.0)	0 (0.0)	0 (0.0)	0 (0.0)	0 (0.0)
Seeds and kernels (n=35	NOVA3 (n=18)	0 (0.0)	0 (0.0)	0 (0.0)	0 (0.0)	0 (0.0)	0 (0.0)	0 (0.0)	0 (0.0)	0 (0.0)	0 (0.0)	0 (0.0)	0 (0.0)	0 (0.0)	0 (0.0)
	NOVA4 (n=17)	1 (5.9)	0 (0.0)	0 (0.0)	0 (0.0)	2 (11.8)	0 (0.0)	0 (0.0)	0 (0.0)	0 (0.0)	0 (0.0)	0 (0.0)	0 (0.0)	0 (0.0)	0 (0.0)
Nut or seed product (n=27)	NOVA1 (n=9)	0 (0.0)	0 (0.0)	0 (0.0)	0 (0.0)	0 (0.0)	0 (0.0)	9 (100.0)	0 (0.0)	0 (0.0)	0 (0.0)	0 (0.0)	0 (0.0)	0 (0.0)	0 (0.0)

	NOVA4 (n=18)	3 (16.7)	8 (44.4)	6 (33.3)	0 (0.0)	5 (27.8)	4 (22.2)	16 (88.9)	0 (0.0)	3 (16.7)	0 (0.0)	0 (0.0)	0 (0.0)	0 (0.0)	0 (0.0)
Vegetable (excluding potato) (n=170)	NOVA1 (n=65)	0 (0.0)	0 (0.0)	0 (0.0)	0 (0.0)	0 (0.0)	0 (0.0)	0 (0.0)	0 (0.0)	0 (0.0)	0 (0.0)	0 (0.0)	0 (0.0)	1 (1.5)	0 (0.0)
	NOVA3 (n=35)	1 (2.9)	0 (0.0)	0 (0.0)	0 (0.0)	1 (2.9)	0 (0.0)	0 (0.0)	1 (2.9)	1 (2.9)	0 (0.0)	0 (0.0)	0 (0.0)	0 (0.0)	0 (0.0)
	NOVA4 (n=70)	3 (4.3)	1 (1.4)	0 (0.0)	0 (0.0)	0 (0.0)	0 (0.0)	0 (0.0)	0 (0.0)	4 (5.7)	0 (0.0)	0 (0.0)	0 (0.0)	2 (2.9)	0 (0.0)
Starchy root or potato (n=21)	NOVA1 (n=1)	0 (0.0)	0 (0.0)	0 (0.0)	0 (0.0)	0 (0.0)	0 (0.0)	0 (0.0)	0 (0.0)	0 (0.0)	0 (0.0)	0 (0.0)	0 (0.0)	0 (0.0)	0 (0.0)
	NOVA3 (n=2)	0 (0.0)	0 (0.0)	0 (0.0)	0 (0.0)	0 (0.0)	0 (0.0)	0 (0.0)	0 (0.0)	0 (0.0)	0 (0.0)	0 (0.0)	0 (0.0)	0 (0.0)	0 (0.0)
	NOVA4 (n=18)	10 (55.6)	6 (33.3)	0 (0.0)	3 (16.7)	0 (0.0)	0 (0.0)	0 (0.0)	0 (0.0)	5 (27.8)	0 (0.0)	0 (0.0)	0 (0.0)	3 (16.7)	0 (0.0)
Pulse and pulse product (n=341)	NOVA1 (n=341)	0 (0.0)	122 (35.8)	79 (23.2)	0 (0.0)	90 (26.4)	12 (3.5)	81 (23.8)	0 (0.0)	0 (0.0)	0 (0.0)	0 (0.0)	0 (0.0)	5 (1.5)	0 (0.0)
Processed food product (fruit) (n=42)	NOVA1 (n=1)	0 (0.0)	0 (0.0)	0 (0.0)	0 (0.0)	0 (0.0)	0 (0.0)	0 (0.0)	0 (0.0)	1 (100)	0 (0.0)	0 (0.0)	0 (0.0)	0 (0.0)	0 (0.0)
	NOVA3 (n=5)	0 (0.0)	0 (0.0)	0 (0.0)	0 (0.0)	0 (0.0)	0 (0.0)	0 (0.0)	0 (0.0)	0 (0.0)	0 (0.0)	0 (0.0)	0 (0.0)	0 (0.0)	0 (0.0)
	NOVA4 (n=36)	0 (0.0)	4 (11.1)	0 (0.0)	0 (0.0)	4 (11.1)	4 (11.1)	2 (5.6)	0 (0.0)	15 (41.7)	0 (0.0)	0 (0.0)	0 (0.0)	0 (0.0)	0 (0.0)
Sugar, honey, or syrup (n=46)	NOVA1 (n=1)	0 (0.0)	0 (0.0)	0 (0.0)	0 (0.0)	0 (0.0)	0 (0.0)	0 (0.0)	0 (0.0)	0 (0.0)	0 (0.0)	0 (0.0)	0 (0.0)	0 (0.0)	0 (0.0)
	NOVA2 (n=35)	0 (0.0)	0 (0.0)	0 (0.0)	0 (0.0)	0 (0.0)	0 (0.0)	0 (0.0)	0 (0.0)	0 (0.0)	0 (0.0)	0 (0.0)	0 (0.0)	0 (0.0)	0 (0.0)
	NOVA3 (n=6)	0 (0.0)	0 (0.0)	0 (0.0)	0 (0.0)	0 (0.0)	0 (0.0)	0 (0.0)	0 (0.0)	0 (0.0)	0 (0.0)	0 (0.0)	0 (0.0)	0 (0.0)	0 (0.0)
	NOVA4 (n=4)	0 (0.0)	0	0 (0.0)	0 (0.0)	0 (0.0)	0 (0.0)	0 (0.0)	0 (0.0)	0 (0.0)	0 (0.0)	0 (0.0)	0 (0.0)	0 (0.0)	0 (0.0)
Jam or marmalade (n=83)	NOVA4 (n=83)	3 (6.5)	11 (13.3)	0 (0.0)	1 (1.2)	1 (1.2)	0 (0.0)	0 (0.0)	0 (0.0)	4 (4.8)	0 (0.0)	0 (0.0)	0 (0.0)	0 (0.0)	0 (0.0)
Non-chocolate confectionery	NOVA3 (n=22)	0 (0.0)	0 (0.0)	0 (0.0)	0 (0.0)	9 (40.9)	6 (27.3)	8 (36.4)	0 (0.0)	1 (4.5)	0 (0.0)	0 (0.0)	0 (0.0)	0 (0.0)	0 (0.0)

or other sugar product (n=68)	NOVA4 (n=46)	3 (6.5)	3 (6.5)	3 (6.5)	5 (10.9)	36 (78.3)	29 (63.0)	34 (73.9)	0 (0.0)	10 (21.7)	0 (0.0)	0 (0.0)	0 (0.0)	0 (0.0)	0 (0.0)
Chocolate or chocolate product (n=207)	NOVA4 (n=207)	207 (100.0)	161 (77.8)	185 (89.4)	28 (13.5)	196 (94.7)	76 (36.7)	14 (6.8)	0 (0.0)	6 (2.9)	0 (0.0)	0 (0.0)	0 (0.0)	0 (0.0)	0 (0.0)
Juice or nectar (n=163)	NOVA1 (n=48)	0	0 (0.0)	0 (0.0)	0 (0.0)	0 (0.0)	0 (0.0)	0 (0.0)	0 (0.0)	0 (0.0)	0 (0.0)	0 (0.0)	0 (0.0)	0 (0.0)	0 (0.0)
	NOVA3 (n=1)	0 (0.0)	0 (0.0)	0 (0.0)	0 (0.0)	0 (0.0)	0 (0.0)	0 (0.0)	0 (0.0)	0 (0.0)	0 (0.0)	0 (0.0)	0 (0.0)	1 (100)	0 (0.0)
	NOVA4 (n=114)	0 (0.0)	0	0	0 (0.0)	0 (0.0)	0 (0.0)	0 (0.0)	0 (0.0)	2 (1.8)	0 (0.0)	0 (0.0)	0 (0.0)	0 (0.0)	0 (0.0)
Non-alcoholic beverage (n=283)	NOVA4 (n=283)	15 (5.3)	5 (1.8)	14 (4.9)	0 (0.0)	3 (1.1)	1 (0.4)	1 (0.4)	0 (0.0)	0 (0.0)	0 (0.0)	0 (0.0)	0 (0.0)	0 (0.0)	0 (0.0)
Spice, Condiment or other Ingredient (n=282)	NOVA1 (n=1)	0	0 (0.0)	0 (0.0)	0 (0.0)	0 (0.0)	0 (0.0)	0 (0.0)	0 (0.0)	0 (0.0)	0 (0.0)	0 (0.0)	0 (0.0)	0 (0.0)	0 (0.0)
	NOVA2 (n=1)	0 (0.0)	0 (0.0)	0 (0.0)	0 (0.0)	1 (100.0)	1 (100)	1 (100.0)	0 (0.0)	0 (0.0)	0 (0.0)	0 (0.0)	0 (0.0)	0 (0.0)	0 (0.0)
	NOVA3 (n=7)	0	0 (0.0)	0 (0.0)	0 (0.0)	1 (14.3)	0 (0.0)	0 (0.0)	1 (14.3)	0 (0.0)	0 (0.0)	0 (0.0)	0 (0.0)	0 (0.0)	0 (0.0)
	NOVA4 (n=273)	110 (40.3)	118 (43.2)	80 (29.3)	97 (35.5)	51 (18.7)	22 (8.1)	17 (6.2)	90 (33.0)	30 (11.0)	28 (10.3)	7 (2.6)	1 (0.4)	65 (23.8)	1 (0.4)
Prepared food product (n=164)	NOVA4 (n=164)	124 (75.6)	111 (67.7)	73 (44.5)	53 (32.3)	18 (11.0)	17 (10.4)	19 (11.6)	50 (30.5)	5 (3.0)	39 (23.8)	2 (1.2)	0 (0.0)	73 (44.5)	13 (7.9)
Ready-to-eat Food (n=38)	NOVA4 (n=38)	26 (68.4)	31 (81.6)	22 (57.9)	22 (57.9)	9 (23.7)	7 (18.4)	12 (31.6)	23 (60.5)	23 (60.5)	19 (50.0)	7 (18.4)	11 (28.9)	32 (84.5)	0 (0.0)
Frozen, Semi-Ready Meal (n=40)	NOVA4 (n=40)	9 (22.5)	17 (42.5)	8 (20.0)	7 (17.5)	1 (2.5)	0 (0.0)	6 (15.0)	4 (10.0)	5 (12.5)	1 (2.5)	0 (0.0)	3 (7.5)	12 (30.0)	0 (0.0)

Table S2 Prevalence of the 14 allergens in the products' ingredient list per NOVA Group of each food subcategory.

Food Subcategory	NOVA Group	Milk	Gluten	Soy	Eggs	Nuts	Peanuts	Sesame	Mustard	Sulphites	Fish	Crustaceans	Mollusks	Celery	Lupin
Milk (n=171)	NOVA1 (n=94)	94 (100.0)	0 (0.0)	0 (0.0)	0 (0.0)	0 (0.0)	0 (0.0)	0 (0.0)	0 (0.0)	0 (0.0)	0 (0.0)	0 (0.0)	0 (0.0)	0 (0.0)	0 (0.0)
	NOVA3 (n=6)	6 (100)	0 (0.0)	0 (0.0)	0 (0.0)	0 (0.0)	0 (0.0)	0 (0.0)	0 (0.0)	0 (0.0)	0 (0.0)	0 (0.0)	0 (0.0)	0 (0.0)	0 (0.0)
	NOVA4 (n=71)	71 (100.0)	0 (0.0)	3 (4.2)	0 (0.0)	0 (0.0)	0 (0.0)	0 (0.0)	0 (0.0)	0 (0.0)	0 (0.0)	0 (0.0)	0 (0.0)	0 (0.0)	0 (0.0)
Yogurt (n=168)	NOVA1 (n=46)	46 (100.0)	0 (0.0)	0 (0.0)	0 (0.0)	0 (0.0)	0 (0.0)	0 (0.0)	0 (0.0)	0 (0.0)	0 (0.0)	0 (0.0)	0 (0.0)	0 (0.0)	0 (0.0)
	NOVA4 (n=122)	122 (100.0)	23 (18.9)	6 (4.9)	3 (2.5)	8 (6.6)	0 (0.0)	0 (0.0)	0 (0.0)	0 (0.0)	0 (0.0)	0 (0.0)	0 (0.0)	0 (0.0)	0 (0.0)
Cheese (n=209)	NOVA3 (n=124)	124 (100.0)	0 (0.0)	0 (0.0)	0 (0.0)	0 (0.0)	0 (0.0)	0 (0.0)	0 (0.0)	0 (0.0)	0 (0.0)	0 (0.0)	0 (0.0)	0 (0.0)	0 (0.0)
	NOVA4 (n=85)	85 (100.0)	3 (3.5)	0 (0.0)	1 (1.2)	0 (0.0)	0 (0.0)	0 (0.0)	0 (0.0)	1 (1.2)	1 (1.2)	0 (0.0)	0 (0.0)	1 (1.2)	0 (0.0)
Milk Substitute (n=334)	NOVA1 (n=6)	0 (0.0)	1 (16.7)	3 (50.0)	0 (0.0)	2 (33.3)	0 (0.0)	0 (0.0)	0 (0.0)	0 (0.0)	0 (0.0)	0 (0.0)	0 (0.0)	0 (0.0)	0 (0.0)
	NOVA3 (n=37)	0 (0.0)	13 (35.1)	5 (13.5)	0 (0.0)	14 (37.8)	0 (0.0)	0 (0.0)	0 (0.0)	0 (0.0)	0 (0.0)	0 (0.0)	0 (0.0)	0 (0.0)	0 (0.0)
	NOVA4 (n=291)	5 (1.7)	47 (16.2)	62 (21.3)	0 (0.0)	98 (33.7)	3 (1.0)	4 (1.4)	0 (0.0)	0 (0.0)	0 (0.0)	0 (0.0)	0 (0.0)	4 (1.4)	0 (0.0)
Milk Cream (n=40)	NOVA4 (n=40)	40 (100.0)	0 (0.0)	2 (5.0)	0 (0.0)	0 (0.0)	0 (0.0)	0 (0.0)	0 (0.0)	0 (0.0)	0 (0.0)	0 (0.0)	0 (0.0)	0 (0.0)	0 (0.0)
Dairy Dessert (n=43)	NOVA4 (n=43)	41 (95.3)	8 (18.6)	24 (55.8)	6 (14.0)	15 (34.9)	0 (0.0)	0 (0.0)	0 (0.0)	0 (0.0)	0 (0.0)	0 (0.0)	0 (0.0)	0 (0.0)	0 (0.0)
Fresh or processed eggs (n=35)	NOVA1 (n=34)	3 (8.8)	1 (2.9)	1 (2.9)	34 (100.0)	1 (2.9)	0 (0.0)	0 (0.0)	0 (0.0)	0 (0.0)	0 (0.0)	0 (0.0)	0 (0.0)	0 (0.0)	0 (0.0)
	NOVA4 (n=1)	0 (0.0)	0 (0.0)	0 (0.0)	1 (100.0)	0 (0.0)	0 (0.0)	0 (0.0)	0 (0.0)	0 (0.0)	0 (0.0)	0 (0.0)	0 (0.0)	0 (0.0)	0 (0.0)
Egg imitation (n=1)	NOVA4 (n=1)	0 (0.0)	0 (0.0)	1 (100.0)	0 (0.0)	0 (0.0)	0 (0.0)	0 (0.0)	0 (0.0)	0 (0.0)	0 (0.0)	0 (0.0)	0 (0.0)	0 (0.0)	0 (0.0)
Poultry meat (n=2)	NOVA4 (n=2)	2 (100.0)	0 (0.0)	0 (0.0)	0 (0.0)	0 (0.0)	0 (0.0)	0 (0.0)	0 (0.0)	0 (0.0)	0 (0.0)	0 (0.0)	0 (0.0)	0 (0.0)	0 (0.0)
Meat analogue (n=110)	NOVA4 (n=110)	0 (0.0)	64 (58.2)	44 (40.0)	0 (0.0)	0 (0.0)	0 (0.0)	1 (0.9)	6 (5.5)	3 (2.7)	0 (0.0)	0 (0.0)	0 (0.0)	1 (0.9)	0 (0.0)
Preserved meat (n=82)	NOVA1 (n=1)	1 (100.0)	0 (0.0)	1 (100.0)	0 (0.0)	0 (0.0)	1 (100)	0 (0.0)	1 (100)	0 (0.0)	0 (0.0)	0 (0.0)	0 (0.0)	1 (100)	0 (0.0)
	NOVA3 (n=2)	0 (0.0)	0 (0.0)	0 (0.0)	0 (0.0)	0 (0.0)	0 (0.0)	0 (0.0)	0 (0.0)	0 (0.0)	0 (0.0)	0 (0.0)	0 (0.0)	0 (0.0)	0 (0.0)
	NOVA4 (n=79)	34 (43.0)	6 (7.6)	22 (27.8)	1 (1.3)	0 (0.0)	4 (5.1)	0 (0.0)	9 (11.4)	2 (2.5)	0 (0.0)	0 (0.0)	0 (0.0)	7 (8.9)	0 (0.0)

Sausage or similar meat (n=37)	NOVA4 (n=37)	13 (35.1)	2 (5.4)	11 (29.7)	0 (0.0)	0 (0.0)	1 (2.7)	0 (0.0)	5 (13.5)	2 (5.4)	0 (0.0)	0 (0.0)	0 (0.0)	1 (2.7)	0 (0.0)
Meat dish (n=17)	NOVA4 (n=17)	8 (47.1)	14 (82.4)	5 (29.4)	4 (23.5)	0 (0.0)	1 (5.9)	0 (0.0)	1 (5.9)	0 (0.0)	0 (0.0)	0 (0.0)	0 (0.0)	2 (11.8)	0 (0.0)
Seafood product (n=78)	NOVA3 (n=52)	0 (0.0)	0 (0.0)	2 (3.8)	0 (0.0)	0 (0.0)	0 (0.0)	0 (0.0)	0 (0.0)	0 (0.0)	50 (96.2)	0 (0.0)	2 (3.8)	3 (5.8)	0 (0.0)
	NOVA4 (n=26)	1 (3.8)	9 (34.6)	1 (3.8)	0 (0.0)	0 (0.0)	0 (0.0)	0 (0.0)	1 (3.8)	2 (7.7)	19 (73.1)	1 (3.8)	7 (26.9)	0 (0.0)	0 (0.0)
Vegetable fat or oil (n=8)	NOVA2 (n=1)	0 (0.0)	0 (0.0)	0 (0.0)	0 (0.0)	0 (0.0)	0 (0.0)	0 (0.0)	0 (0.0)	0 (0.0)	0 (0.0)	0 (0.0)	0 (0.0)	0 (0.0)	0 (0.0)
	NOVA4 (n=7)	3 (42.9)	0	0 (0.0)	0 (0.0)	0 (0.0)	0 (0.0)	0 (0.0)	0 (0.0)	0 (0.0)	0 (0.0)	0 (0.0)	0 (0.0)	0 (0.0)	0 (0.0)
Margarine or lipid of mixed origins (n=39)	NOVA4 (n=39)	21 (53.8)	0 (0.0)	1 (2.6)	0 (0.0)	0 (0.0)	0 (0.0)	0 (0.0)	0 (0.0)	0 (0.0)	0 (0.0)	0 (0.0)	0 (0.0)	0 (0.0)	0 (0.0)
Butter or other animal fat (n=34)	NOVA2 (n=32)	32 (100.0)	0 (0.0)	0 (0.0)	0 (0.0)	0 (0.0)	0 (0.0)	0 (0.0)	0 (0.0)	0 (0.0)	0 (0.0)	0 (0.0)	0 (0.0)	0 (0.0)	0 (0.0)
	NOVA4 (n=2)	2 (100.0)	0 (0.0)	0 (0.0)	0 (0.0)	0 (0.0)	0 (0.0)	0 (0.0)	0 (0.0)	0 (0.0)	0 (0.0)	0 (0.0)	0 (0.0)	0 (0.0)	0 (0.0)
Cereal or cereal-like milling products (n=51)	NOVA4 (n=51)	11 (21.6)	51 (100.0)	12 (23.5)	7 (13.7)	0 (0.0)	0 (0.0)	0 (0.0)	0 (0.0)	0 (0.0)	0 (0.0)	0 (0.0)	0 (0.0)	0 (0.0)	0 (0.0)
Rice or other grain (n=97)	NOVA1 (n=64)	0 (0.0)	2 (3.1)	0 (0.0)	0 (0.0)	0 (0.0)	0 (0.0)	0 (0.0)	0 (0.0)	0 (0.0)	0 (0.0)	0 (0.0)	0 (0.0)	0 (0.0)	0 (0.0)
	NOVA3 (n=2)	0 (0.0)	0 (0.0)	0 (0.0)	0 (0.0)	0 (0.0)	0 (0.0)	0 (0.0)	0 (0.0)	0 (0.0)	0 (0.0)	0 (0.0)	0 (0.0)	0 (0.0)	0 (0.0)
	NOVA4 (n=31)	7 (22.6)	25 (80.6)	14 (45.2)	5 (16.1)	0 (0.0)	0 (0.0)	0 (0.0)	3 (9.7)	0 (0.0)	1 (3.2)		0 (0.0)	10 (32.3)	0 (0.0)
Pasta and similar products (n=200)	NOVA1 (n=165)	5 (3.0)	161 (98.2)	1 (0.6)	10 (6.1)	0 (0.0)	0 (0.0)	0 (0.0)	0 (0.0)	0 (0.0)	0 (0.0)	0 (0.0)	0 (0.0)	0 (0.0)	0 (0.0)
	NOVA3 (n=9)	8 (88.9)	9 (100.0)	0 (0.0)	2 (22.2)	0 (0.0)	0 (0.0)	0 (0.0)	0 (0.0)	0 (0.0)	0 (0.0)	0 (0.0)	0 (0.0)	0 (0.0)	0 (0.0)
	NOVA4 (n=26)	16 (61.5)	22 (84.6)	0 (0.0)	16 (61.5)	0 (0.0)	0 (0.0)	0 (0.0)	0 (0.0)	0 (0.0)	0 (0.0)	0 (0.0)	0 (0.0)	0 (0.0)	0 (0.0)
Breakfast cereals (n=149)	NOVA1 (n=4)	0 (0.0)	4 (100.0)	0 (0.0)	0 (0.0)	0 (0.0)	0 (0.0)	0 (0.0)	0 (0.0)	0 (0.0)	0 (0.0)	0 (0.0)	0 (0.0)	0 (0.0)	0 (0.0)
	NOVA3 (n=1)	0 (0.0)	1 (100.0)	0 (0.0)	0 (0.0)	0 (0.0)	0 (0.0)	0 (0.0)	0 (0.0)	0 (0.0)	0 (0.0)	0 (0.0)	0 (0.0)	0 (0.0)	0 (0.0)
	NOVA4 (n=144)	63 (43.8)	132 (91.7)	66 (45.8)	0 (0.0)	26 (18.1)	11 (7.6)	2 (1.4)	0 (0.0)	12 (8.3)	0 (0.0)	0 (0.0)	0 (0.0)	0 (0.0)	0 (0.0)
Bread and similar products (n=242)	NOVA4 (n=242)	33 (13.6)	236 (97.5)	32 (13.2)	9 (3.7)	1 (0.4)	0 (0.0)	47 (19.4)	3 (1.2)	4 (1.7)	0 (0.0)	0 (0.0)	0 (0.0)	1 (0.4)	1 (0.4)
Fine bakery ware (n=279)	NOVA4 (n=279)	189 (67.7)	277 (99.3)	155 (55.6)	91 (32.6)	43 (15.4)	3 (1.1)	9 (3.2)	0 (0.0)	7 (2.5)	0 (0.0)	0 (0.0)	0 (0.0)	0 (0.0)	3 (1.1)

Savory cereal dish (n=86)	NOVA4 (n=86)	82 (95.3)	86 (100.0)	21 (24.4)	31 (36.0)	0 (0.0)	1 (1.2)	4 (4.7)	2 (2.3)	0 (0.0)	0 (0.0)	0 (0.0)	0 (0.0)	3 (3.5)	0 (0.0)
Nuts (n=65)	NOVA1 (n=20)	0 (0.0)	1 (5.0)	0 (0.0)	0 (0.0)	15 (75.0)	0 (0.0)	0 (0.0)	0 (0.0)	0 (0.0)	0 (0.0)	0 (0.0)	0 (0.0)	0 (0.0)	0 (0.0)
	NOVA3 (n=14)	0 (0.0)	1 (7.1)	0 (0.0)	0 (0.0)	9 (64.3)	6 (42.9)	0 (0.0)	0 (0.0)	0 (0.0)	0 (0.0)	0 (0.0)	0 (0.0)	0 (0.0)	0 (0.0)
	NOVA4 (n=31)	2 (6.5)	17 (54.8)	4 (12.9)	0 (0.0)	16 (51.6)	16 (51.6)	1 (3.2)	0 (0.0)	4 (12.9)	0 (0.0)	0 (0.0)	0 (0.0)	0 (0.0)	0 (0.0)
Seeds and kernels (n=35	NOVA3 (n=18)	0 (0.0)	0 (0.0)	0 (0.0)	0 (0.0)	0 (0.0)	0 (0.0)	0 (0.0)	0 (0.0)	0 (0.0)	0 (0.0)	0 (0.0)	0 (0.0)	0 (0.0)	0 (0.0)
	NOVA4 (n=17)	1 (5.9)	0 (0.0)	0 (0.0)	0 (0.0)	0 (0.0)	0 (0.0)	0 (0.0)	0 (0.0)	0 (0.0)	0 (0.0)	0 (0.0)	0 (0.0)	0 (0.0)	0 (0.0)
Nut or seed product (n=27)	NOVA1 (n=9)	0 (0.0)	0 (0.0)	0 (0.0)	0 (0.0)	0 (0.0)	0 (0.0)	9 (100.0)	0 (0.0)	0 (0.0)	0 (0.0)	0 (0.0)	0 (0.0)	0 (0.0)	0 (0.0)
	NOVA4 (n=18)	1 (5.6)	0	6 (33.3)	0 (0.0)	2 (11.1)	4 (22.2)	12 (66.7)	0 (0.0)	0 (0.0)	0 (0.0)	0 (0.0)	0 (0.0)	0 (0.0)	0 (0.0)
Vegetable (excluding potato) (n=170)	NOVA1 (n=65)	0 (0.0)	0 (0.0)	0 (0.0)	0 (0.0)	0 (0.0)	0 (0.0)	0 (0.0)	0 (0.0)	0 (0.0)	0 (0.0)	0 (0.0)	0 (0.0)	0 (0.0)	0 (0.0)
	NOVA3 (n=35)	1 (2.9)	0 (0.0)	0 (0.0)	0 (0.0)	0 (0.0)	0 (0.0)	0 (0.0)	1 (2.9)	0 (0.0)	0 (0.0)	0 (0.0)	0 (0.0)	0 (0.0)	0 (0.0)
	NOVA4 (n=70)	3 (4.3)	1 (1.4)	0 (0.0)	0 (0.0)	0 (0.0)	0 (0.0)	0 (0.0)	0 (0.0)	3 (4.7)	0 (0.0)	0 (0.0)	0 (0.0)	0 (0.0)	0 (0.0)
Starchy root or potato (n=21)	NOVA1 (n=1)	0 (0.0)	0 (0.0)	0 (0.0)	0 (0.0)	0 (0.0)	0 (0.0)	0 (0.0)	0 (0.0)	0 (0.0)	0 (0.0)	0 (0.0)	0 (0.0)	0 (0.0)	0 (0.0)
	NOVA3 (n=2)	0 (0.0)	0 (0.0)	0 (0.0)	0 (0.0)	0 (0.0)	0 (0.0)	0 (0.0)	0 (0.0)	0 (0.0)	0 (0.0)	0 (0.0)	0 (0.0)	0 (0.0)	0 (0.0)
	NOVA4 (n=18)	2 (11.1)	0	0 (0.0)	0 (0.0)	0 (0.0)	0 (0.0)	0 (0.0)	0 (0.0)	5 (27.8)	0 (0.0)	0 (0.0)	0 (0.0)	0 (0.0)	0 (0.0)
Pulse and pulse product (n=341)	NOVA1 (n=341)	0 (0.0)	1 (0.3)	0 (0.0)	0 (0.0)	0 (0.0)	0 (0.0)	0 (0.0)	0 (0.0)	0 (0.0)	0 (0.0)	0 (0.0)	0 (0.0)	0 (0.0)	0 (0.0)
Processed food product (fruit) (n=42)	NOVA1 (n=1)	0 (0.0)	0 (0.0)	0 (0.0)	0 (0.0)	0 (0.0)	0 (0.0)	0 (0.0)	0 (0.0)	1 (100)	0 (0.0)	0 (0.0)	0 (0.0)	0 (0.0)	0 (0.0)
	NOVA3 (n=5)	0 (0.0)	0 (0.0)	0 (0.0)	0 (0.0)	0 (0.0)	0 (0.0)	0 (0.0)	0 (0.0)	0 (0.0)	0 (0.0)	0 (0.0)	0 (0.0)	0 (0.0)	0 (0.0)
	NOVA4 (n=36)	0 (0.0)	0	0 (0.0)	0 (0.0)	0 (0.0)	0 (0.0)	0 (0.0)	0 (0.0)	14 (38.9)	0 (0.0)	0 (0.0)	0 (0.0)	0 (0.0)	0 (0.0)
Sugar, honey or syrup (n=46)	NOVA1 (n=1)	0 (0.0)	0 (0.0)	0 (0.0)	0 (0.0)	0 (0.0)	0 (0.0)	0 (0.0)	0 (0.0)	0 (0.0)	0 (0.0)	0 (0.0)	0 (0.0)	0 (0.0)	0 (0.0)
	NOVA2 (n=35)	0 (0.0)	0 (0.0)	0 (0.0)	0 (0.0)	0 (0.0)	0 (0.0)	0 (0.0)	0 (0.0)	0 (0.0)	0 (0.0)	0 (0.0)	0 (0.0)	0 (0.0)	0 (0.0)
	NOVA3 (n=6)	0 (0.0)	0 (0.0)	0 (0.0)	0 (0.0)	0 (0.0)	0 (0.0)	0 (0.0)	0 (0.0)	0 (0.0)	0 (0.0)	0 (0.0)	0 (0.0)	0 (0.0)	0 (0.0)
	NOVA4 (n=4)	0 (0.0)	0 (0.0)	0 (0.0)	0 (0.0)	0 (0.0)	0 (0.0)	0 (0.0)	0 (0.0)	0 (0.0)	0 (0.0)	0 (0.0)	0 (0.0)	0 (0.0)	0 (0.0)
Jam or marmalade (n=83)	NOVA4 (n=83)	1 (1.2)	2 (2.4)	0 (0.0)	1 (1.2)	1 (1.2)	0 (0.0)	0 (0.0)	0 (0.0)	0 (0.0)	0 (0.0)	0 (0.0)	0 (0.0)	0 (0.0)	0 (0.0)
	NOVA3 (n=22)	0 (0.0)	0 (0.0)	0 (0.0)	0 (0.0)	2 (9.1)	1 (4.5)	7 (31.8)	0 (0.0)	1 (4.5)	0 (0.0)	0 (0.0)	0 (0.0)	0 (0.0)	0 (0.0)

Non-chocolate confectionery or other sugar product (n=68)	NOVA4 (n=46)	1 (2.2)	1 (2.2)	2 (4.3)	3 (6.5)	12 (26.1)	2 (4.3)	31 (67.4)	0 (0.0)	5 (10.9)	0 (0.0)	0 (0.0)	0 (0.0)	0 (0.0)	0 (0.0)
Chocolate or chocolate product (n=207)	NOVA4 (n=207)	159 (76.8)	49 (23.7)	182 (87.9)	1 (0.5)	75 (36.2)	8 (3.9)	1 (0.5)	0 (0.0)	1 (0.5)	0 (0.0)	0 (0.0)	0 (0.0)	0 (0.0)	0 (0.0)
Juice or nectar (n=163)	NOVA1 (n=48)	0	0 (0.0)	0 (0.0)	0 (0.0)	0 (0.0)	0 (0.0)	0 (0.0)	0 (0.0)	0 (0.0)	0 (0.0)	0 (0.0)	0 (0.0)	0 (0.0)	0 (0.0)
	NOVA3 (n=1)	0 (0.0)	0 (0.0)	0 (0.0)	0 (0.0)	0 (0.0)	0 (0.0)	0 (0.0)	0 (0.0)	0 (0.0)	0 (0.0)	0 (0.0)	0 (0.0)	1 (100)	0 (0.0)
	NOVA4 (n=114)	0 (0.0)	0	0 (0.0)	0 (0.0)	0 (0.0)	0 (0.0)	0 (0.0)	0 (0.0)	2 (1.8)	0 (0.0)	0 (0.0)	0 (0.0)	0 (0.0)	0 (0.0)
Non-alcoholic beverage (n=283)	NOVA4 (n=283)	5 (1.8)	2 (0.7)	10 (3.5)	0 (0.0)	0 (0.0)	0 (0.0)	0 (0.0)	0 (0.0)	0 (0.0)	0 (0.0)	0 (0.0)	0 (0.0)	0 (0.0)	0 (0.0)
Spice, Condiment or other Ingredient (n=282)	NOVA1 (n=1)	0	0 (0.0)	0 (0.0)	0 (0.0)	0 (0.0)	0 (0.0)	0 (0.0)	0 (0.0)	0 (0.0)	0 (0.0)	0 (0.0)	0 (0.0)	0 (0.0)	0 (0.0)
	NOVA2 (n=1)	0	0 (0.0)	0 (0.0)	0 (0.0)	0 (0.0)	0 (0.0)	1 (100.0)	0 (0.0)	0 (0.0)	0 (0.0)	0 (0.0)	0 (0.0)	0 (0.0)	0 (0.0)
	NOVA3 (n=7)	0	0 (0.0)	0 (0.0)	0 (0.0)	0 (0.0)	0 (0.0)	0 (0.0)	1 (14.3)	0 (0.0)	0 (0.0)	0 (0.0)	0 (0.0)	0 (0.0)	0 (0.0)
	NOVA4 (n=273)	60 (22.0)	65 (23.8)	33 (12.1)	43 (15.8)	7 (2.6)	1 (0.4)	1 (0.4)	59 (21.6)	23 (8.4)	3 (1.1)	0 (0.0)	1 (0.4)	25 (9.2)	0 (0.0)
Prepared food product (n=164)	NOVA4 (n=164)	80 (48.8)	55 (33.5)	22 (13.4)	29 (17.7)	1 (0.6)	1 (0.6)	4 (2.4)	24 (14.6)	3 (1.8)	7 (4.3)	2 (1.2)	0 (0.0)	18 (11.0)	0 (0.0)
Ready-to-eat Food (n=38)	NOVA4 (n=38)	19 (50.0)	25 (65.8)	11 (28.9)	15 (39.5)	3 (7.9)	1 (2.6)	2 (5.3)	12 (31.6)	17 (44.7)	5 (13.2)	2 (5.3)	1 (2.6)	8 (21.1)	0 (0.0)
Frozen, Semi-Ready Meal (n=40)	NOVA4 (n=40)	6(15.0)	16 (40.0)	8 (20.0)	2 (5.0)	1 (2.5)	0 (0.0)	1 (2.5)	0 (0.0)	0 (0.0)	1 (2.5)	0 (0.0)	0 (0.0)	3 (7.5)	0 (0.0)

Table S3 Prevalence of the 14 allergens as traces in a precautionary statement per NOVA Group of each food subcategory.

Food Subcategory	NOVA Group	Milk	Gluten	Soy	Eggs	Nuts	Peanuts	Sesame	Mustard	Sulphites	Fish	Crustaceans	Mollusks	Celery	Lupin
Milk (n=171)	NOVA1 (n=94)	0 (0.0)	0 (0.0)	0 (0.0)	0 (0.0)	0 (0.0)	0 (0.0)	0 (0.0)	0 (0.0)	0 (0.0)	0 (0.0)	0 (0.0)	0 (0.0)	0 (0.0)	0 (0.0)
	NOVA3 (n=6)	0 (0.0)	0 (0.0)	0 (0.0)	0 (0.0)	0 (0.0)	0 (0.0)	0 (0.0)	0 (0.0)	0 (0.0)	0 (0.0)	0 (0.0)	0 (0.0)	0 (0.0)	0 (0.0)
	NOVA4 (n=71)	0 (0.0)	0 (0.0)	0 (0.0)	0 (0.0)	0 (0.0)	0 (0.0)	0 (0.0)	0 (0.0)	0 (0.0)	0 (0.0)	0 (0.0)	0 (0.0)	0 (0.0)	0 (0.0)
Yogurt(n=168)	NOVA1 (n=46)	0 (0.0)	0 (0.0)	0 (0.0)	0 (0.0)	0 (0.0)	0 (0.0)	0 (0.0)	0 (0.0)	0 (0.0)	0 (0.0)	0 (0.0)	0 (0.0)	0 (0.0)	0 (0.0)
	NOVA4 (n=122)	0 (0.0)	9 (7.4)	4 (3.3)	8 (6.6)	13 (10.7)	7 (5.7)	10 (8.2)	0 (0.0)	0 (0.0)	0 (0.0)	0 (0.0)	0 (0.0)	0 (0.0)	1 (0.8)
Cheese (n=209)	NOVA3 (n=124)	0 (0.0)	0 (0.0)	0 (0.0)	0 (0.0)	0 (0.0)	0 (0.0)	0 (0.0)	0 (0.0)	0 (0.0)	0 (0.0)	0 (0.0)	0 (0.0)	0 (0.0)	0 (0.0)
	NOVA4 (n=85)	0 (0.0)	1 (1.2)	3 (3.5)	6 (7.1)	0 (0.0)	0 (0.0)	0 (0.0)	4 (4.7)	4 (4.7)	0 (0.0)	0 (0.0)	0 (0.0)	3 (3.5)	0 (0.0)
Milk Imitations (n=334)	NOVA1 (n=6)	0 (0.0)	0 (0.0)	0 (0.0)	0 (0.0)	2 (33.3)	0 (0.0)	0 (0.0)	0 (0.0)	0 (0.0)	0 (0.0)	0 (0.0)	0 (0.0)	0 (0.0)	0 (0.0)
	NOVA3 (n=37)	0 (0.0)	0 (0.0)	2 (5.4)	0 (0.0)	1 (16.7)	0 (0.0)	0 (0.0)	0 (0.0)	0 (0.0)	0 (0.0)	0 (0.0)	0 (0.0)	0 (0.0)	0 (0.0)
	NOVA4 (n=291)	9 (3.1)	9 (3.1)	19 (6.5)	8 (2.7)	48 (16.5)	5 (1.7)	27 (9.3)	16 (5.5)	0 (0.0)	0 (0.0)	0 (0.0)	0 (0.0)	12 (4.1)	0 (0.0)
Milk Cream (n=40)	NOVA4 (n=40)	0 (0.0)	0 (0.0)	0 (0.0)	0 (0.0)	0 (0.0)	0 (0.0)	0 (0.0)	0 (0.0)	0 (0.0)	0 (0.0)	0 (0.0)	0 (0.0)	0 (0.0)	0 (0.0)
Dairy Dessert (n=43)	NOVA4 (n=43)	0 (0.0)	15 (34.9)	10 (23.3)	18 (41.9)	20 (46.5)	0 (0.0)	4 (9.3)	0 (0.0)	0 (0.0)	0 (0.0)	0 (0.0)	0 (0.0)	0 (0.0)	0 (0.0)
Fresh or processed eggs (n=35)	NOVA1 (n=34)	0 (0.0)	2 (5.9)	2 (5.9)	0 (0.0)	2 (5.9)	0 (0.0)	0 (0.0)	0 (0.0)	0 (0.0)	0 (0.0)	0 (0.0)	0 (0.0)	0 (0.0)	0 (0.0)
	NOVA4 (n=1)	0 (0.0)	0 (0.0)	0 (0.0)	0 (0.0)	0 (0.0)	0 (0.0)	0 (0.0)	0 (0.0)	0 (0.0)	0 (0.0)	0 (0.0)	0 (0.0)	0 (0.0)	0 (0.0)
Egg imitation (n=1)	NOVA4 (n=1)	0 (0.0)	0 (0.0)	0 (0.0)	0 (0.0)	0 (0.0)	0 (0.0)	1 (100.0)	1 (100)	0 (0.0)	0 (0.0)	0 (0.0)	0 (0.0)	1 (100)	0 (0.0)
Poultry meat (n=2)	NOVA4 (n=2)	0 (0.0)	0 (0.0)	0 (0.0)	0 (0.0)	0 (0.0)	0 (0.0)	0 (0.0)	0 (0.0)	0 (0.0)	0 (0.0)	0 (0.0)	0 (0.0)	0 (0.0)	0 (0.0)

Meat analogue (n=110)	NOVA4 (n=110)	0 (0.0)	8 (7.3)	15 (13.6)	3 (2.7)	10 (9.1)	0 (0.0)	17 (15.5)	37 (33.6)	0 (0.0)	0 (0.0)	0 (0.0)	0 (0.0)	38 (34.5)	4 (3.6)
Preserved meat (n=82)	NOVA1 (n=1)	0 (0.0)	0 (0.0)	0 (0.0)	1 (100.0)	1 (100.0)	0 (0.0)	0 (0.0)	0 (0.0)	1 (100)	0 (0.0)	0 (0.0)	0 (0.0)	0 (0.0)	0 (0.0)
	NOVA3 (n=2)	0 (0.0)	0 (0.0)	0 (0.0)	0 (0.0)	0 (0.0)	0 (0.0)	0 (0.0)	0 (0.0)	0 (0.0)	0 (0.0)	0 (0.0)	0 (0.0)	0 (0.0)	0 (0.0)
	NOVA4 (n=79)	20 (25.3)	11 (13.9)	34 (43.0)	28 (35.4)	41 (51.9)	6 (7.6)	1 (1.3)	48 (60.8)	26 (32.9)	0 (0.0)	0 (0.0)	0 (0.0)	45 (57.0)	0 (0.0)
Sausage or similar meat (n=37)	NOVA4 (n=37)	8 (21.6)	4 (10.8)	9 (24.3)	13 (35.1)	11 (29.7)	4 (10.8)	0 (0.0)	15 (40.5)	10 (27.0)	0 (0.0)	0 (0.0)	0 (0.0)	18 (48.6)	0 (0.0)
Meat dish (n=17)	NOVA4 (n=17)	7 (41.2)	1 (5.9)	0 (0.0)	1 (5.9)	1 (5.9)	0 (0.0)	0 (0.0)	7 (41.2)	0 (0.0)	0 (0.0)	0 (0.0)	0 (0.0)	0 (0.0)	0 (0.0)
Seafood product (n=78)	NOVA3 (n=52)	0 (0.0)	1 (1.9)	0 (0.0)	0 (0.0)	0 (0.0)	0 (0.0)	0 (0.0)	0 (0.0)	0 (0.0)	0 (0.0)	1 (1.9)	0 (0.0)	0 (0.0)	0 (0.0)
	NOVA4 (n=26)	12 (30.8)	1 (3.8)	1 (3.8)	1 (3.8)	0 (0.0)	0 (0.0)	0 (0.0)	1 (3.8)	0 (0.0)	0 (0.0)	0 (0.0)	2 (7.7)	1 (3.8)	0 (0.0)
Vegetable fat or oil (n=8)	NOVA2 (n=1)	0 (0.0)	0 (0.0)	0 (0.0)	0 (0.0)	0 (0.0)	0 (0.0)	0 (0.0)	0 (0.0)	0 (0.0)	0 (0.0)	0 (0.0)	0 (0.0)	0 (0.0)	0 (0.0)
	NOVA4 (n=7)	0 (0.0)	0 (0.0)	0 (0.0)	0 (0.0)	0 (0.0)	0 (0.0)	0 (0.0)	0 (0.0)	0 (0.0)	0 (0.0)	0 (0.0)	0 (0.0)	0 (0.0)	0 (0.0)
Margarine or lipid of mixed origins (n=39)	NOVA4 (n=39)	12 (30.8)	0 (0.0)	0 (0.0)	0 (0.0)	0 (0.0)	0 (0.0)	0 (0.0)	0 (0.0)	0 (0.0)	0 (0.0)	0 (0.0)	0 (0.0)	0 (0.0)	0 (0.0)
Butter or other animal fat (n=34)	NOVA2 (n=32)	0 (0.0)	0 (0.0)	0 (0.0)	0 (0.0)	0 (0.0)	0 (0.0)	0 (0.0)	0 (0.0)	0 (0.0)	0 (0.0)	0 (0.0)	0 (0.0)	0 (0.0)	0 (0.0)
	NOVA4 (n=2)	0 (0.0)	0 (0.0)	0 (0.0)	0 (0.0)	0 (0.0)	0 (0.0)	0 (0.0)	0 (0.0)	0 (0.0)	0 (0.0)	0 (0.0)	0 (0.0)	0 (0.0)	0 (0.0)
Cereal or cereal-like milling products and derivatives (n=51)	NOVA4 (n=51)	25 (49.0)	0 (0.0)	15 (29.4)	28 (54.9)	15 (29.4)	4 (7.8)	21 (41.2)	16 (31.4)	1 (2.0)	1 (2.0)	1 (2.0)	1 (2.0)	5 (9.8)	0 (0.0)
Rice or other grain (n=97)	NOVA1 (n=64)	0 (0.0)	1 (1.6)	1 (1.6)	1 (1.6)	5 (7.8)	5 (7.8)	5 (7.8)	0 (0.0)	0 (0.0)	0 (0.0)	0 (0.0)	0 (0.0)	0 (0.0)	0 (0.0)

	NOVA3 (n=2)	0 (0.0)	0 (0.0)	0 (0.0)	0 (0.0)	0 (0.0)	0 (0.0)	0 (0.0)	0 (0.0)	0 (0.0)	0 (0.0)	0 (0.0)	0 (0.0)	0 (0.0)	0 (0.0)
	NOVA4 (n=31)	12 (38.7)	3 (9.7)	8 (25.8)	16 (51.6)	0 (0.0)	5 (16.1)	13 (41.9)	16 (51.6)	0 (0.0)	6 (19.4)	0 (0.0)	1 (3.2)	9 (29.0)	7 (22.6)
Pasta and similar products (n=200)	NOVA1 (n=165)	9 (5.5)	0 (0.0)	63 (38.2)	42 (25.5)	1 (0.6)	1 (0.6)	1 (0.6)	1 (0.6)	0 (0.0)	0 (0.0)	0 (0.0)	0 (0.0)	1 (0.6)	1 (0.6)
	NOVA3 (n=9)	0 (0.0)	0 (0.0)	0 (0.0)	4 (44.4)	0 (0.0)	0 (0.0)	3 (33.3)	0 (0.0)	0 (0.0)	1 (11.1)	1 (11.1)	0 (0.0)	0 (0.0)	0 (0.0)
	NOVA4 (n=26)	4 (15.4)	0 (0.0)	11 (42.3)	3 (11.5)	0 (0.0)	0 (0.0)	0 (0.0)	1 (3.8)	0 (0.0)	0 (0.0)	0 (0.0)	0 (0.0)	1 (3.8)	3 (11.5)
Breakfast cereals (n=149)	NOVA1 (n=4)	1 (25.0)	0 (0.0)	4 (100.0)	0 (0.0)	1 (25.0)	0 (0.0)	1 (25.0)	0 (0.0)	1 (25.0)	0 (0.0)	0 (0.0)	0 (0.0)	0 (0.0)	0 (0.0)
	NOVA3 (n=1)	0 (0.0)	0 (0.0)	1 (100.0)	0 (0.0)	1 (100.0)	1 (100)	0 (0.0)	0 (0.0)	0 (0.0)	0 (0.0)	0 (0.0)	0 (0.0)	0 (0.0)	0 (0.0)
	NOVA4 (n=144)	54 (37.5)	3 (2.1)	28 (19.4)	3 (2.1)	86 (59.7)	45 (31.3)	24 (16.7)	2 (1.4)	0 (0.0)	0 (0.0)	0 (0.0)	0 (0.0)	0 (0.0)	2 (1.4)
Bread and similar products (n=242)	NOVA4 (n=242)	88 (35.4)	2 (0.8)	41 (16.9)	79 (32.6)	46 (19.0)	4 (1.7)	102 (42.1)	1 (0.4)	0 (0.0)	0 (0.0)	0 (0.0)	0 (0.0)	1 (0.4)	2 (0.8)
Fine bakery ware (n=279)	NOVA4 (n=279)	51 (18.3)	0 (0.0)	60 (21.5)	106 (38.0)	174 (62.4)	65 (23.3)	137 (49.1)	5 (1.8)	13 (4.7)	0 (0.0)	0 (0.0)	0 (0.0)	5 (1.8)	17 (6.1)
Savory cereal dish (n=86)	NOVA4 (n=86)	3 (3.5)	0 (0.0)	24 (27.9)	36 (41.9)	37 (43.0)	1 (1.2)	61 (70.9)	38 (44.2)	2 (2.3)	2 (2.3)	0 (0.0)	0 (0.0)	21 (24.4)	1 (1.2)
Nuts (n=65)	NOVA1 (n=20)	0 (0.0)	18 (90.0)	1 (5.0)	0 (0.0)	5 (25.0)	18 (90.0)	13 (65.0)	0 (0.0)	0 (0.0)	0 (0.0)	0 (0.0)	0 (0.0)	0 (0.0)	0 (0.0)
	NOVA3 (n=14)	0 (0.0)	9 (64.3)	0 (0.0)	0 (0.0)	4 (28.6)	8 (57.1)	10 (71.4)	0 (0.0)	0 (0.0)	0 (0.0)	0 (0.0)	0 (0.0)	0 (0.0)	0 (0.0)
	NOVA4 (n=31)	2 (6.5)	11 (35.5)	1 (3.2)	0 (0.0)	15 (48.4)	14 (45.2)	20 (64.5)	1 (3.2)	0 (0.0)	0 (0.0)	0 (0.0)	0 (0.0)	0 (0.0)	0 (0.0)
Seeds and kernels (n=35)	NOVA3 (n=18)	0 (0.0)	0 (0.0)	0 (0.0)	0 (0.0)	0 (0.0)	0 (0.0)	0 (0.0)	0 (0.0)	0 (0.0)	0 (0.0)	0 (0.0)	0 (0.0)	0 (0.0)	0 (0.0)
	NOVA4 (n=17)	0 (0.0)	0 (0.0)	0 (0.0)	0 (0.0)	2 (11.8)	0 (0.0)	0 (0.0)	0 (0.0)	0 (0.0)	0 (0.0)	0 (0.0)	0 (0.0)	0 (0.0)	0 (0.0)
	NOVA1 (n=9)	0 (0.0)	0 (0.0)	0 (0.0)	0 (0.0)	0 (0.0)	0 (0.0)	0 (0.0)	0 (0.0)	0 (0.0)	0 (0.0)	0 (0.0)	0 (0.0)	0 (0.0)	0 (0.0)

Nut or seed product (n=27)	NOVA4 (n=18)	2 (11.1)	8 (44.4)	0 (0.0)	0 (0.0)	3 (16.7)	0 (0.0)	4 (22.2)	0 (0.0)	3 (16.7)	0 (0.0)	0 (0.0)	0 (0.0)	0 (0.0)	0 (0.0)
Vegetable (excluding potato) (n=170)	NOVA1 (n=65)	0 (0.0)	0 (0.0)	0 (0.0)	0 (0.0)	0 (0.0)	0 (0.0)	0 (0.0)	0 (0.0)	0 (0.0)	0 (0.0)	0 (0.0)	0 (0.0)	1 (1.5)	0 (0.0)
	NOVA3 (n=35)	0 (0.0)	0 (0.0)	0 (0.0)	0 (0.0)	1 (2.9)	0 (0.0)	0 (0.0)	0 (0.0)	1 (2.9)	0 (0.0)	0 (0.0)	0 (0.0)	0 (0.0)	0 (0.0)
	NOVA4 (n=70)	0 (0.0)	0 (0.0)	0 (0.0)	0 (0.0)	0 (0.0)	0 (0.0)	0 (0.0)	0 (0.0)	1 (1.4)	0 (0.0)	0 (0.0)	0 (0.0)	2 (2.9)	0 (0.0)
Starchy root or potato (n=21)	NOVA1 (n=1)	0 (0.0)	0 (0.0)	0 (0.0)	0 (0.0)	0 (0.0)	0 (0.0)	0 (0.0)	0 (0.0)	0 (0.0)	0 (0.0)	0 (0.0)	0 (0.0)	0 (0.0)	0 (0.0)
	NOVA3 (n=2)	0 (0.0)	0 (0.0)	0 (0.0)	0 (0.0)	0 (0.0)	0 (0.0)	0 (0.0)	0 (0.0)	0 (0.0)	0 (0.0)	0 (0.0)	0 (0.0)	0 (0.0)	0 (0.0)
	NOVA4 (n=18)	8 (44.4)	6 (33.3)	0 (0.0)	3 (16.7)	0 (0.0)	0 (0.0)	0 (0.0)	0 (0.0)	0 (0.0)	0 (0.0)	0 (0.0)	0 (0.0)	3 (16.7)	0 (0.0)
Pulse and pulse product (n=341)	NOVA1 (n=341)	0 (0.0)	121 (35.5)	79 (23.2)	0 (0.0)	90 (26.4)	12 (3.5)	81 (23.8)	0 (0.0)	0 (0.0)	0 (0.0)	0 (0.0)	0 (0.0)	5 (1.5)	0 (0.0)
Processed food product (fruit) (n=42)	NOVA1 (n=1)	0 (0.0)	0 (0.0)	0 (0.0)	0 (0.0)	0 (0.0)	0 (0.0)	0 (0.0)	0 (0.0)	0 (0.0)	0 (0.0)	0 (0.0)	0 (0.0)	0 (0.0)	0 (0.0)
	NOVA3 (n=5)	0 (0.0)	0 (0.0)	0 (0.0)	0 (0.0)	0 (0.0)	0 (0.0)	0 (0.0)	0 (0.0)	0 (0.0)	0 (0.0)	0 (0.0)	0 (0.0)	0 (0.0)	0 (0.0)
	NOVA4 (n=36)	0 (0.0)	4 (11.1)	0 (0.0)	0 (0.0)	4 (11.1)	4 (11.1)	2 (5.6)	0 (0.0)	1 (2.8)	0 (0.0)	0 (0.0)	0 (0.0)	0 (0.0)	0 (0.0)
Sugar, honey or syrup (n=46)	NOVA1 (n=1)	0 (0.0)	0 (0.0)	0 (0.0)	0 (0.0)	0 (0.0)	0 (0.0)	0 (0.0)	0 (0.0)	0 (0.0)	0 (0.0)	0 (0.0)	0 (0.0)	0 (0.0)	0 (0.0)
	NOVA2 (n=35)	0 (0.0)	0 (0.0)	0 (0.0)	0 (0.0)	0 (0.0)	0 (0.0)	0 (0.0)	0 (0.0)	0 (0.0)	0 (0.0)	0 (0.0)	0 (0.0)	0 (0.0)	0 (0.0)
	NOVA3 (n=6)	0 (0.0)	0 (0.0)	0 (0.0)	0 (0.0)	0 (0.0)	0 (0.0)	0 (0.0)	0 (0.0)	0 (0.0)	0 (0.0)	0 (0.0)	0 (0.0)	0 (0.0)	0 (0.0)
	NOVA4 (n=4)	0 (0.0)	0 (0.0)	0 (0.0)	0 (0.0)	0 (0.0)	0 (0.0)	0 (0.0)	0 (0.0)	0 (0.0)	0 (0.0)	0 (0.0)	0 (0.0)	0 (0.0)	0 (0.0)
Jam or marmalade (n=83)	NOVA4 (n=83)	2 (2.4)	9 (10.8)	0 (0.0)	0 (0.0)	0 (0.0)	0 (0.0)	0 (0.0)	0 (0.0)	4 (4.8)	0 (0.0)	0 (0.0)	0 (0.0)	0 (0.0)	0 (0.0)
Non-chocolate	NOVA3 (n=22)	0 (0.0)	0 (0.0)	0 (0.0)	0 (0.0)	7 (31.8)	5 (22.7)	1 (4.5)	0 (0.0)	0 (0.0)	0 (0.0)	0 (0.0)	0 (0.0)	0 (0.0)	0 (0.0)

confectionery or other sugar product (n=68)	NOVA4 (n=46)	2 (4.3)	2 (4.3)	1 (2.2)	2 (4.3)	24 (52.2)	27 (58.7)	3 (6.5)	0 (0.0)	5 (10.9)	0 (0.0)	0 (0.0)	0 (0.0)	0 (0.0)	0 (0.0)
Chocolate or chocolate product (n=207)	NOVA4 (n=207)	48 (23.2)	113 (54.6)	3 (1.4)	27 (13.0)	121 (58.5)	68 (32.9)	13 (6.3)	0 (0.0)	5 (2.4)	0 (0.0)	0 (0.0)	0 (0.0)	0 (0.0)	0 (0.0)
Juice or nectar (n=163)	NOVA1 (n=48)	0 (0.0)	0 (0.0)	0 (0.0)	0 (0.0)	0 (0.0)	0 (0.0)	0 (0.0)	0 (0.0)	0 (0.0)	0 (0.0)	0 (0.0)	0 (0.0)	0 (0.0)	0 (0.0)
	NOVA3 (n=1)	0 (0.0)	0 (0.0)	0 (0.0)	0 (0.0)	0 (0.0)	0 (0.0)	0 (0.0)	0 (0.0)	0 (0.0)	0 (0.0)	0 (0.0)	0 (0.0)	0 (0.0)	0 (0.0)
	NOVA4 (n=114)	0 (0.0)	0 (0.0)	0 (0.0)	0 (0.0)	0 (0.0)	0 (0.0)	0 (0.0)	0 (0.0)	0 (0.0)	0 (0.0)	0 (0.0)	0 (0.0)	0 (0.0)	0 (0.0)
Non-alcoholic beverage (n=283)	NOVA4 (n=283)	10 (3.5)	3 (1.1)	4 (1.4)	0 (0.0)	3 (1.1)	1 (0.4)	1 (0.4)	0 (0.0)	0 (0.0)	0 (0.0)	0 (0.0)	0 (0.0)	0 (0.0)	0 (0.0)
Spice, Condiment or other Ingredient (n=282)	NOVA1 (n=1)	0 (0.0)	0 (0.0)	0 (0.0)	0 (0.0)	0 (0.0)	0 (0.0)	0 (0.0)	0 (0.0)	0 (0.0)	0 (0.0)	0 (0.0)	0 (0.0)	0 (0.0)	0 (0.0)
	NOVA2 (n=1)	0 (0.0)	0 (0.0)	0 (0.0)	0 (0.0)	1 (100.0)	1 (100)	0 (0.0)	0 (0.0)	0 (0.0)	0 (0.0)	0 (0.0)	0 (0.0)	0 (0.0)	0 (0.0)
	NOVA3 (n=7)	0 (0.0)	0 (0.0)	0 (0.0)	0 (0.0)	1 (14.3)	0 (0.0)	0 (0.0)	0 (0.0)	0 (0.0)	0 (0.0)	0 (0.0)	0 (0.0)	0 (0.0)	0 (0.0)
	NOVA4 (n=273)	50 (18.3)	53 (19.4)	47 (17.2)	54 (19.8)	44 (16.1)	21 (7.7)	16 (5.9)	31 (11.4)	7 (2.6)	25 (9.2)	7 (2.6)	0 (0.0)	40 (14.7)	1 (0.4)
Prepared food product (n=164)	NOVA4 (n=164)	45 (27.4)	56 (34.1)	51 (31.1)	24 (14.6)	17 (10.4)	16 (9.8)	15 (9.1)	26 (15.9)	2 (1.2)	32 (19.5)	0 (0.0)	0 (0.0)	55 (33.5)	13 (7.9)
Ready-to-eat Food (n=38)	NOVA4 (n=38)	7 (18.4)	6 (15.8)	11 (28.9)	7 (18.4)	6 (15.8)	6 (15.8)	10 (26.3)	11 (28.9)	6 (15.8)	14 (36.8)	5 (13.2)	10 (26.3)	24 (63.2)	0 (0.0)
Frozen, Semi-Ready Meal (n=40)	NOVA4 (n=40)	3 (7.5)	1 (2.5)	0 (0.0)	5 (12.5)	0 (0.0)	0 (0.0)	5 (12.5)	4 (10.0)	5 (12.5)	0 (0.0)	0 (0.0)	3 (7.5)	8 (20.0)	0 (0.0)
